# Redox Polymers for Tissue Engineering

**DOI:** 10.3389/fmedt.2021.669763

**Published:** 2021-05-24

**Authors:** Binbin Z. Molino, Junji Fukuda, Paul J. Molino, Gordon G. Wallace

**Affiliations:** ^1^Faculty of Engineering, Yokohama National University, Yokohama, Japan; ^2^Kanagawa Institute of Industrial Science and Technology, Kawasaki, Japan; ^3^Australian Research Council (ARC) Centre of Excellence for Electromaterials Science, Intelligent Polymer Research Institute, University of Wollongong, Wollongong, NSW, Australia

**Keywords:** conducting polymer, graphene, poly(3,4-ethylenedioxythiophene), tissue engineering, biofabrication, polypyrrole, polyaniline

## Abstract

This review will focus on the targeted design, synthesis and application of redox polymers for use in regenerative medicine and tissue engineering. We define redox polymers to encompass a variety of polymeric materials, from the multifunctional conjugated conducting polymers to graphene and its derivatives, and have been adopted for use in the engineering of several types of stimulus responsive tissues. We will review the fundamental properties of organic conducting polymers (OCPs) and graphene, and how their properties are being tailored to enhance material - biological interfacing. We will highlight the recent development of high-resolution 3D fabrication processes suitable for biomaterials, and how the fabrication of intricate scaffolds at biologically relevant scales is providing exciting opportunities for the application of redox polymers for both *in-vitro* and *in-vivo* tissue engineering. We will discuss the application of OCPs in the controlled delivery of bioactive compounds, and the electrical and mechanical stimulation of cells to drive behaviour and processes towards the generation of specific functional tissue. We will highlight the relatively recent advances in the use of graphene and the exploitation of its physicochemical and electrical properties in tissue engineering. Finally, we will look forward at the future of organic conductors in tissue engineering applications, and where the combination of materials development and fabrication processes will next unite to provide future breakthroughs.

## Introduction

Over the past several years the field of tissue engineering has seen enormous advancements, with the development of novel materials and fabrication methodologies promising to open new avenues and opportunities for the design and construction of the next generation of scaffolds and conduits that address the many biological processes required for the engineering of complex, functional tissue. Several biomaterial properties generally need to be considered to provide an optimal scaffold system for interfacing with- and guiding *in-vitro* and *in-vivo* biological interactions and processes. Broadly speaking, these include (i) the tailoring of material physicochemical and mechanical properties, (ii) the incorporation and effective delivery of bioactive chemical agents; and (iii) engagement of fabrication methodologies that allow the construction of complex and biologically relevant architectures in which morphological and chemical cues may be spatially arranged throughout the 3D scaffold.

However, while the above approaches provide physicochemical, mechanical, chemical and morphological cues, they lack the ability to provide a fundamental stimulus that has been shown critical in guiding cell behaviour and biological properties critical to engineering of new, functional tissues: *electrical stimuli*. Galvani was first to report of the use of an external electrical stimulus to promote a biological response in the late 1790's, however it has not been until recent decades that the true potential of delivering electrical signals to cells and tissues has been realised. Perhaps the most famous application has been the advent of the cochlear implant, that provides the ability for those with profound hearing impairment to once again hear, thanks to the translation of acoustic stimuli into an electrical signal, that is subsequently delivered to the excitable auditory neuronal cells in the cochlear *via* a micro-platinum electrode array (1). The application of electrical stimuli to a range of cell types has since been shown to promote several significant biological processes (i.e., cell adhesion, proliferation, alignment, and differentiation) (2–5), providing enormous potential in the field of tissue engineering. It has however become clear that the use of hard, metallic electrodes is unsuitable for the majority of tissue engineering applications due to their high modulus, low processability, non-degradable nature and high risk of initiating faradaic processes at elevated charge injection levels.

The discovery of organic conducting polymers (OCPs) in the late 1970s by Shirakawa, MacDiarmid, and Heeger (6, 7), for which they would later win the Nobel Prize in chemistry (2000), immediately spawned a new field of research that caught the attention of those in academia and industry, with initial applications including flexible electronics, light-emitting diodes and antistatic coatings (8). It was not long before the potential use of these materials in biology was realised (9), with the field growing significantly over the past 2 decades based primarily on the inherent biocompatibility nature of OCPs, and the capacity to improve and tailor their physicochemical, electrical and mechanical properties by tuning polymer chemistry and through electrochemical control. This has led to potential applications in biosensing (10), bio-batteries (11, 12), drug delivery (13), tissue engineering (14, 15) and electrodes for bio-electronics interfaces (16, 17). The more recent discovery of graphene in 2004 (18) further energised researchers in the field of electroactive biomaterials. This provided new opportunities for the application of an organic conductor to interface directly with biomolecules, cells and tissues, providing unique physicochemical, mechanical and electrical properties, that along with a high level of processability and facile approaches for chemical functionalisation, has promised to open significant opportunities for its use in regenerative medicine (19).

This review will present the development and application of the redox active biomaterials OCPs and graphene for tissue engineering applications. It will highlight their advantages over traditional conductors, and how applying clever materials processing and fabrication technologies are allowing the fabrication of intricate biomaterial scaffold architectures that present several critical physicochemical, chemical, topographical, mechanical and electrical properties that are essential for the generation of complex, functional tissues. Finally, we will look ahead to the future, where advances in materials development and processing, together with advancements in fabrication, will deliver the next generation of redox polymer-based technologies in regenerative medicine.

## Organic Conductors

### OCPs

**Figure 1:**
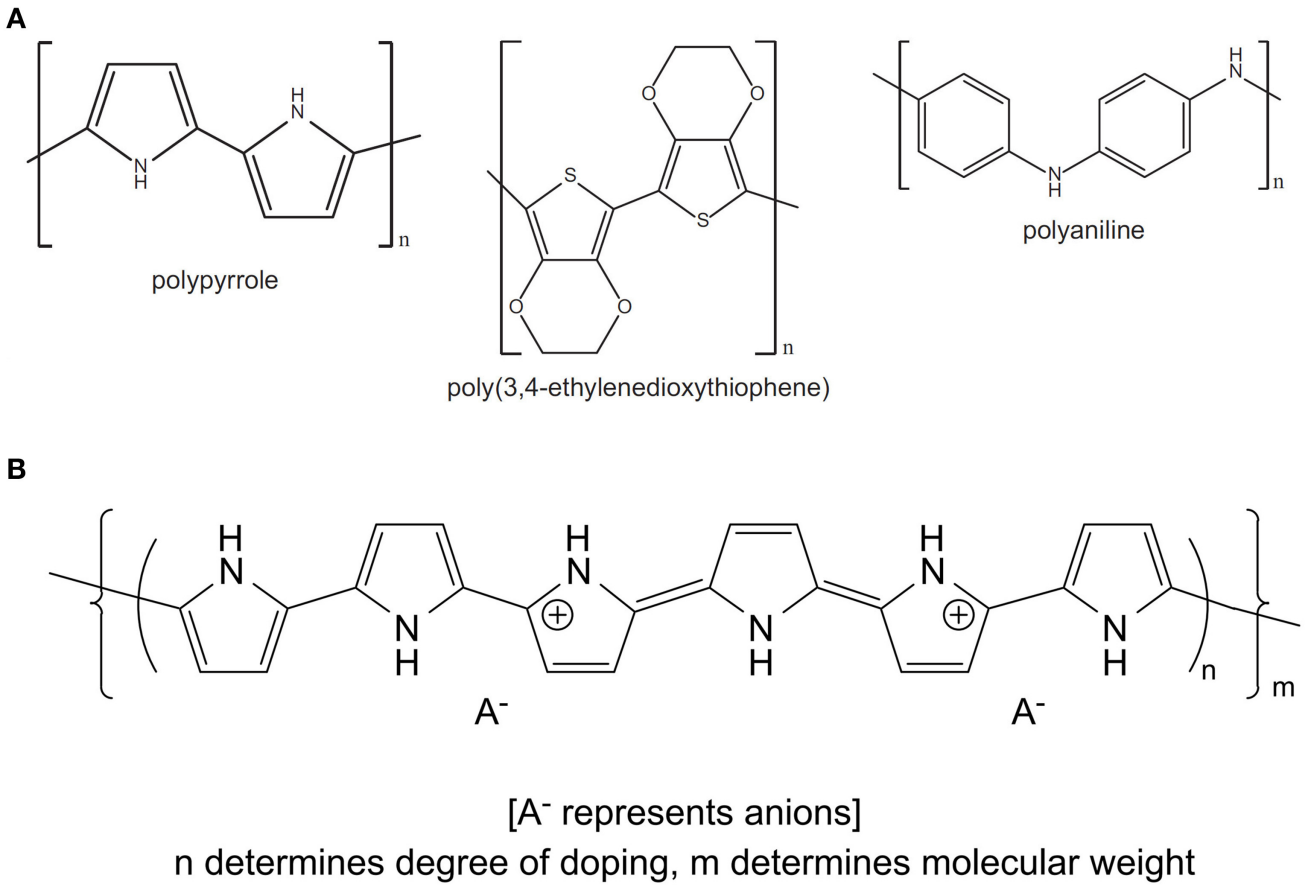
**(A)** Chemical structures of the common OCPs polypyrrole, poly(3,4-ethylenedioxythiophene) and polyaniline. **(B)** Schematic illustrating the incorporation of a dopant anion during oxidation of monomeric species to form the OCP conjugated backbone (PPy). **(B)** Reproduced with permission from Gilmore et al. (27).

OCPs, including polypyrrole (PPy), polyaniline (PANI) and poly(3,4-ethylenedioxythiophene) (PEDOT), are an exciting class of materials that are biocompatible, and shown to perform numerous biologically relevant functions including guided cell growth (20), controlled drug delivery (21), electrical stimulation (22, 23) and recording (24), and mechanical (25) stimulation. OCPs are conjugated polymers, where oxidation of the monomeric unit (either electrochemically or chemically) generates a positively charged polymer backbone that is counterbalanced by the incorporation of an anionic species, termed the *dopant*, in a process known as *doping* (26) (Figure 1). The physicochemical, electrochemical and mechanical properties of OCP materials, including conductivity, modulus, surface energy, and nanotopography, can be tailored by varying the identity of the monomer or dopant species, the method of polymerisation (i.e., vapour phase, chemical or electrochemical) and the synthesis conditions (e.g., temperature, pH, solvent, dopant concentration, etc.) (26, 28–31). Electrochemical polymerisation is limited to the deposition of OCP surface coatings on conducting substrates and therefore is incompatible with the development of composites or materials that can be easily processed further. Chemical synthesis on the other hand allows the development of OCP nanoparticles and dispersions that can be easily blended with secondary polymers, and/or fabricated using techniques such as spray coating, dip coating, ink-jet printing, wetspinning, electrospinning, and extrusion printing to form complex scaffold structures better suited to development of scaffolds and conduits for biological applications. Vapour phase polymerisation provides an approach for the deposition of homogenous films on both conductive and non-conductive substrates (32, 33).

Perhaps the most common approach to modulate fundamental OCP properties is *via* the choice of the dopant anion. Traditionally, small synthetic dopants have been used to optimise polymer electroactivity, conductivity and stability. The most commonly incorporated synthetic dopants include polystyrene sulfonate (PSS), chloride, dodecylbenzenesulfonic acid (DBSA) and para(toluene sulfonic acid) (pTS), and have been employed to develop OCP biomaterials that have shown good biocompatibility, interfacing effectively with a range of biomolecules, proteins, cells and tissues. An approach many researchers have pursued to improve the biocompatibility of OCP materials has been to engage biomolecules as the dopant species. Biomolecules including proteins and peptide fragments, polysaccharides, glycosaminoglycans, amino acids, antibiotics and anti-inflammatories have all been incorporated into OCP materials with the aim of imparting specific biofunctionality to the biomaterial either through presentation of the immobilised biocompound at the OCP interface, or release of the compound *via* passive or electrochemical processes [for review, see (34)]. Recently, the incorporation of large, polyelectrolyte biological dopants such as alginate (35, 36) and Ulvan (36, 37) (an algal derived glycan-extract), have been shown to dramatically influence polymer wettability, shear modulus and surface morphology, with PEDOT-Ulvan polymers shown to support the proliferation of human dermal fibroblasts (37), and the proliferation and differentiation of PC12 neuronal cells with and without electrical stimulation (36).

### Graphene

**Figure 2:**
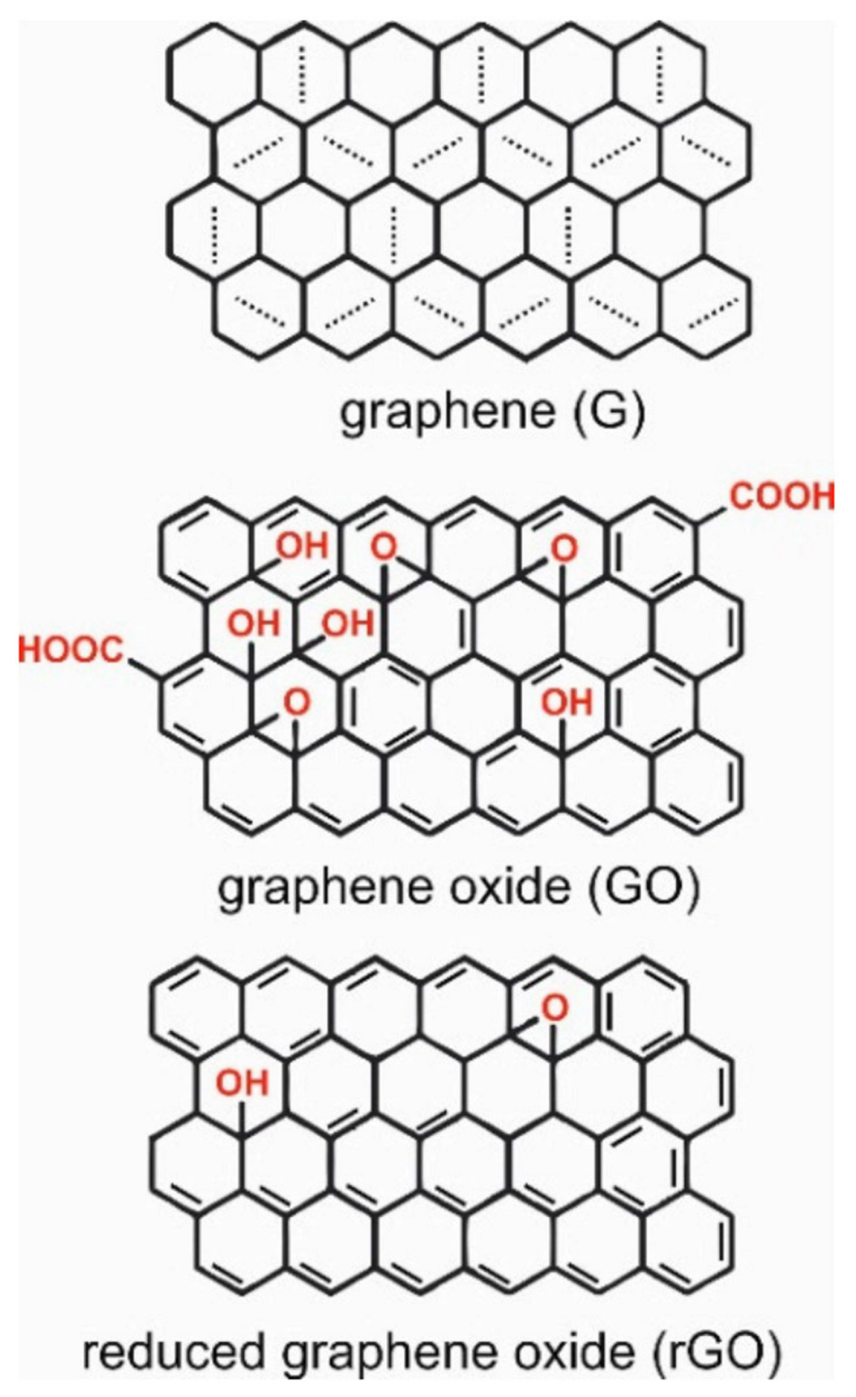
Structures of garaphene (G), graphene oxide (GO), and reduced graphene oxide (rGO). Reproduced with permission from Tadyszak et al. (38).

The relatively recent discovery of graphene has not impeded its rapid identification and application to use in the field of biomedicine, and more specifically tissue engineering and as a conduit for electronic communication with biological systems. Graphene is fundamentally a 2-dimensional hexagonal lattice of carbon atoms, although it may be synthesised to form one of several different derivatives. The most widely used of these derivatives for biological applications are graphene oxide (GO), and reduced graphene oxide (rGO), which possess carboxyl (-OOH), hydroxyl (-OH) or epoxy (-O) groups that can provide improved processability, ability for the covalent attachment of other functional chemistries, and enhanced biocompatibility (Figure 2) (38).

Initial studies exploring the efficacy of graphene as a biomaterial focused on fabricating surface films using chemical vapour deposition (CVD), generating highly conductive and flexible surface films that presented highly biocompatible properties. The biocompatibility of graphene has in part been proposed to result from beneficial adsorption and interfacing with biomolecules and proteins *via* π-π bonding and electrostatic interactions, conditioning the material interface for cell-surface interactions (39). The development of scalable methods for the chemical exfoliation of graphene and the production of highly processable graphene dispersions (40, 41), saw the investigation of graphene and graphene based composites in biomedical applications explode, finding uses in drug delivery, biosensing, bio-imaging, antimicrobial materials and electroactive materials for tissue engineering and electroceuticals [for review, see (19, 42)]. In composite materials, the ability for graphene to impart electrical conductivity, improved mechanical properties and enhanced biological – material interactions has been a major driver in graphene's incorporation into tissue engineering scaffolds. When taken with its mechanical strength, biocompatibility, low cost and scalability, graphene has maintained the attention of researchers in the biomedical sciences, and therefore continues to be under intense investigation for biological applications.

## Bio Fabrication Processes

The need to develop tools that are capable of processing and fabricating structures at a resolution and complexity relevant for tissue engineering and biomedical applications, and that are compatible with materials properties and requirements, including in some cases the incorporation of living cells within polymeric inks (43), has spawned the field of *Biofabrication*. For graphene, the conventional formation of pristine films using CVD on both 2D and 3D template structures has been demonstrated (44–46), however the ability to exfoliate graphene from graphite into graphene, graphene oxide and reduced graphene oxide has significantly extended the reach of this material, allowing blending with secondary polymers, and the utilisation of commonly employed nano- and micro-scale biofabrication techniques. These include electrospinning (47, 48), capable of producing random- or aligned-nanoscale fibres into mats or scaffolds, providing topographical and morphological cues at a scale matching the extracellular matrix. Wetspinning (49) and templating (50) techniques have been used for form cell guides at the micron-scale, allowing the organisation of cells along single fibres compiled into a more complex conduit, while extrusion printing (51, 52) has allowed the discrete spatial deposition of graphene-based composites customised for particular cell and tissue types.

Similarly, OCPs have several approaches for the fabrication of 2D films [i.e., vapour phase deposition (53), electrochemical polymerisation (36), spin coating (54)], as well as their use in inks and composites for fabrication using electrospinning (55, 56) and wetspinning (57) techniques, as well as 3D printing. Both graphene and OCP materials also have bulk processing methodologies that have been exploited for the fabrication of biomaterial scaffolds. These include the formation of aerogels *via* freeze drying (58, 59), generating a porous hydrogel or sponge material that has shown to have advantageous properties for cell adhesion and tissue development. The development of inks composed of OCPs directly conjugated with highly hydrated and large polyelectrolyte anionic species has also been used to form soft and conductive hydrogel materials that can be formed into discrete scaffolds architectures using 3D extrusion printing (60). An overview of some common fabrication approaches for OCP and graphene biomaterials is provided in Table 1.

**Table 1:** Commonly used fabrication approaches for biomaterials incorporating OCPs and graphene.

**Scaffold/material structure**	**Conductor incorporation method**	**Conducting material**
Films (2D)	Chemical vapour deposition	Graphene (44–46, 61)
	Electrochemical polymerisation	OCPs (27, 35, 36, 62–64)
	Vapour phase polymerisation	OCPs (53)
	Spin coating	OCPs (54)
	Chemical polymerisation	OCPs (65, 66)
	Composite	OCPs (67–69)
Films (3D template)	Chemical vapour deposition	Graphene (44)
	Vapour phase polymerisation	OCPs (70)
Electrospun nanofibrous mats	Composite	OCPs (55, 56, 71–73)
		Graphene (47, 48)
	Vapour phase polymerisation	OCPs (74)
	Chemical polymerisation	OCPs (75–78)
Micron scale fibres	Wet spinning – composite	OCPs (57)
		Graphene (49)
	Moulding	Graphene (50)
Aerogels	Composite	Graphene (79, 80)
	Vapour phase polymerisation	OCPs (81)
	Chemical polymerisation	OCPs (58)
Hydrogel	Composite	Graphene (82, 83)
		OCPs (60)
	Electrochemical polymerisation	OCPs (84)
	Chemical polymerisation	OCPs (85, 86)
3D Extrusion Printed Scaffolds	Composite ink	OCPs (60, 87)
		Graphene (51, 52)

Hereafter we review the application of graphene and OCP based materials for tissue engineering applications. Tailoring of material composition and fabrication approaches are used to optimise the biomaterial properties towards the engineering of several tissue types, including cardiac and skeletal muscle, neural tissue, bone and cartilage, and skin. Tables 2 (OCP) and 3 (graphene) provide summaries of the literature reviewed for each material/tissue engineering application.

**Table 2:** Summary of studies describing conducting polymer biomaterial application for tissue engineering.

**Tissue type**	**Material**	**Structure**	**Conductivity/resistance**	**Biocharacterisation**	**References**
Neural tissue	PEDOT-PSS	Spin coated film	5.8 Ωm^−1^	ReNcellVM cells	(88)
	PEDOT doped with Ulvan, DS, CS, ALG or DBSA	Electrochemical polymerised film	–	PC12 cells	(36)
	PPy-CS-Collagen	Electrochemical polymerised film	–	PC12 cells	(89)
	PPy doped with pts, DBSA, CS, PSS, hyaluronic acid and poly(2- methoxyaniline-5-sulfonic acid) – with NT-3 drug co-dopant	Electrochemical polymerised film	–	SNG	(64)
	PPy-PSS-NGF	Electrochemical polymerised film	9.3 ± 2 S cm^−1^	PC12 cells	(90)
	Poly(glycerol sebacate)—aniline pentamer polymer	Drop casting on micropatterned polyurethane	8.2–8.5 × 10^−5^ S/cm	Schwann cells, PC12 Cells	(91)
	PPy modified PLGA	Electrospun PLGA modified *via* chemically polymerised PPy	–	PC12 and hippocampal cells	(75)
	PPy coated PLCL/SF	Electrospun PLCL/SF modified *via* chemically polymerised PPy	1.36 × 10^−4^ to 8.52 × 10^−6^ S/cm	PC12 and Schwann cells	(77)
	PANI/PCL/Gelatin	Electrospun mat	0.02 × 10^−6^ S	Nerve stem cells	(56)
	PANI/PLL	Electrospun mat	3 × 10^−9^ S	Nerve stem cells	(55)
	PPy-pts modified PLCL/SF	Electrospun nerve conduit	–	*In-vivo implantation*	(92)
	PEDOT/chitosan/gelatin	Hydrogel	3.44 × 10^−2^ to 1.72 × 10^−1^ S cm^−1^	PC12	(58)
	PEDOT-CMC	Hydrogel	4.68 ± 0.28 × 10^−3^ S cm^−1^	PC12	(85)
	Aniline – Genepin hydrogel (loaded with dexamethasone)	Hydrogel	3–7 × 10^−4^ S/cm	PC12	(93)
	PPy-cellulose aerogel	Aerogel	1 × 10^−5^ to 0.08 S cm^−1^	PC12	(81)
	PPy-Collagen	Micron-fibres	–	hMSC	(94)
	PEDOT/agarose	Conduit	–	*In-vivo* implantation	(95)
Cardiac tissue	PANI	Drop cast Film (Conductive and non-conductive form)	2 kΩ resistivity	H9c2	(96)
	PANI-phytic acid	Film on chitosan	35.85 ± 9.40 kilohms per square	*Ex-vivo* heart model	(97)
	PPy-PCL	Composite film	1.00 ± 0.40 kohms cm	HL-1 atrial myocytes	(98)
	PPy/PCL/Gelatin	Electrospun mat	0.013–0.37 mS/cm	Primary cardiac myocytes	(71)
	PANI-PLGA	Electrospun mat	3.1 × 10^−3^ S/cm	Neonatal cardiomyocytes	(99)
	PANI-PLA	Electrospun mat	3.6 ± 0.7 × 10^−6^ S/m to 2.1 ± 0.3 × 10^−5^ S/m	H9c2	(100)
	PPy-chitosan	Hydrogel	–	Neonatal rat cardiomyocytes, *in-vivo* study	(86)
	Chitosan-*graft*-aniline tetramer and dibenzaldehyde-terminated PEG	Hydrogel	~23 × 10^−5^ S/cm	C2C12 and H9c2 cells, *in-vivo* study	(43)
Skin tissue	PPy-pts	Electrochemical polymerised film	4.9 × 10^−1^ to 1.5 × 10^−3^ S/cm	Schwann cells and fibroblasts	(101)
	Poly(terthiophene) – RGD	Electrochemical polymerised film	1.22 ± 0.15 S cm^−1^	Human dermal fibroblasts	(102)
	PPy modified PET fa bric	Chemical polymerisation on fabric	–	Human dermal fibroblasts	(66)
	PPy/PLA	Composite film	10^−3^ S/cm	Human cutaneous fibroblasts	(103)
	PPy/Heparin/PLLA	Composite film	Resistivity 10^2^–10^3^ ohms per square	Human dermal fibrolasts	(104)
	PANI/CPSA/PLCL	Electrospun mat	0.0015–0.0138 S.cm^−1^	Human dermal fibroblasts and NIH-3T3 fibroblasts	(105)
	PANI-chitosan	Electrospun mat	~2.6 × 10^−5^ S/m	Fibroblast cells	(73)
Skeletal muscle tissue	PPy doped with pts, HA, DS, CS, PMAS and DBS	Electrochemical polymerised film	–	Rosa primary myoblasts	(27)
	MWCNT-PPy-pts	Electrochemical polymerised film	–	Rosa primary myoblasts	(106)
	Amino capped aniline trimer/PCL	Co-polymer film	–	C2C12 cells	(107)
	PPy/polyurethane	Composite film	9.95 × 10^−11^ ± 8.03 × 10^−11^ to 2.32 × 10^−6^ ± 2.97 × 10^−7^ S/cm	C2C12 cells	(69)
	Aniline trimer/polyurethane-urea co-polymer	Composite film	10^−6^ S/cm	C2C12	(108)
	PANI/PLCL	Electrospun mat	0.160–0.296 S/cm	C2C12	(109)
	PANI/PCL	Electrospun mat	63.6 ± 6.6 mS cm^−1^	C2C12	(110)
Bone tissue engineering	PANI modified Ti nanotubes	Electrochemical polymerised film	Charge transfer resistance of 172.26 Ohms	MC3T3-E1 cells	(111)
	PANI interdigitated electrodes	Film – interdigitated electrodes	5 × 10^−2^ s/cm	BMSC and MC3T3-E1 cells	(112)
	Aniline trimer/PLA	Composite film	–	C2C12	(113)
	PPy/He/PLLA	Composite membrane	–	Osteoblast-like Saos-2 cells	(114)
	PANI/PLA	Electrospun scaffold	0.004–0.032 S/cm	Bone marrow derived mesenchymal stem cells	(115)
	PPy/SiO2/gelatine/hydroxyapatite	Porous scaffold	–	K7M2WT osteoblast cells	(116)
	PEDOT/PCL	Scaffold	–	MSC	(70)

**Table 3:** Summary of studies describing graphene and graphene oxide biomaterial application for tissue engineering.

**Tissue type**	**Material**	**Structure**	**Conductivity/resistance**	**Biocharacterisation**	**References**
Neural tissue	CVD grown graphene on polyethylene terephthalate	Flat film	–	SHSY5Y cells	(45)
	CVD graphene	Flat film on glass	–	hNSC	(46)
	CVD graphene	Flat film on PMMA	–	hMSC	(61)
	CVD graphene on porous nickel template	Porous scaffold (foam) and 2D films	–	NSC	(44)
	Fluorinated graphene sheets	Graphene sheets on PDMS with microchannels	–	MSC	(117)
	rGO-chitosan composite	rGO-chitosan film	–	hMSC	(118)
	GO-SiO2 NP	Film	–	NSC	(62)
	rGO-collagen	3D acellular dermal matrix – rGO composite	–	MSC	(119)
	rGO	Porous rGO scaffold	~10^−3^ S cm^−1^	*In-vivo* implantation	(120, 121)
	Graphene-PVA:alginate	Electrospun mat	Impedance ~25 Ω	PC12	(47)
	rGO modified PVC	rGO modified PVC electrospun mat	12.5 ± 1.2 S cm ^−1^	Primary motor neurons	(48)
	rGO	Micron fibre	4.64 ± 0.90 S cm^−1^	Embryonic neural progenitor cells, *in-vivo* implantation	(50)
	GO/PCL composite	Tubular scaffold	4.55 × 10^−4^ S/cm^−1^	Schwann cells, *in-vivo* implantation	(122)
	Graphene/PLG composite	Extrusion printed scaffold	~800 S m	hMSC, *in-vivo* implantation	(123)
Cardiac tissue	rGO modified silk/fibroin	rGO modification of electrospun mat	Resistance 4.3 MΩ	Rat cardiomyocytes	(124)
	Graphene/PCL	Electrospun mat	–	Mouse embryonic stem cell derived cardiomyocytes	(125)
	rGO - silver/polyurethane	Electrospun mat	~105 μS/cm	Human cardiac progenitor cells	(126)
	rGO coating on collagen	Porous scaffold	9.1 ± 0.9 × 10^−6^ to 1.2 ± 0.4 × 10^−4^ S/m	HUVEC	(127)
	GO-GelMA	Hydrogel	–	H9c2 cardiomyocytes, *in-vivo* implantation	(128)
	rGO/GelMA	Hydrogel	Impedance ~ 4 kΩ	Rat cardiomyocytes	(82)
Bone tissue	Graphene or GO on PDMS	Film	–	MSC	(129)
	rGO modified titanium	Film	–	MC3T3-E1, *in-vivo* implantation	(130)
	nGO/starch composite	Electrospun mat	–	MG63	(131)
	GO/PLA/nHydroxyapatite	Electrospun mat	–	Saos-2	(132)
	GO-hydroxyapatite composite	Porous scaffold	–	Rat bone mesenchymal stem cells, *in-vivo* implantation	(133)
	GO-chitosan composite	Porous scaffold	–	MC3T3-E1	(134)
	GO/strontium NPs/PCL	Porous scaffold	–	MC3T3-E1	(135)
Skeletal muscle tissue	Thermally reduced GO and GO	Film	–	C2C12	(136)
	Graphene	Film (crumpled)	–	C2C12	(137)
	GO/PCL	Electrospun mat	–	hMSC	(138)
	rGO-polyacrylamide	Hydrogel	1.4 ± 0.4 × 10^−4^ S/cm	C2C12	(139)
	rGO/polydopamine	Aerogel	13.289 S m^−1^	C2C12	(140)
Skin tissue	Reduced claisen graphene (rcG) – peptide modified	Film	–	NIH-3T3 fibroblasts, RAW macrophages	(141)
	GO/PLGA or GO/PLGA/collagen	Electrospun mat	–	HDF	(142)
	CVD graphene on Ni	Foam	–	MSC, *in-vivo* implantation	(143)
	GO modified genipin crosslinked ECM	Sponge	–	L929, *in-vivo* implantation	(144)
Cartilage tissue	Graphene or porous graphene oxide/cell biocomposite	Cell – material composite	–	MSC	(145)
	GO/chitosan	Porous scaffold	–	Human articular chondrocytes	(146)
	GO/methacrylated chondroitin sulphate/poly(ethylene glycol) methyl ether-ϵ-caprolactone-acryloyl chloride)	Porous scaffold	~0.73 S/m	3T3, *in-vivo* implantation	(147)
	GO/collagen/chitosan	Printed hydrogel scaffold	–	Chondrocytes, *in-vivo* implantation	(148)
	GO/GelMA/ PEGDA	Printed hydrogel scaffold	–	MSC	(52)

## Organic Conducting Polymers

### Neural Tissue Engineering

2D films have been used to demonstrated OCPs to be highly biocompatible materials that support neural cell adhesion, proliferation and differentiation for a range of neuronal cell types. Crosslinked PEDOT-PSS films were shown to present excellent biocompatibility, increasing the proliferation of fibroblast cells by 1.3 times over 4 days of cell culture, relative to a glass control (88), and later used for the electrical stimulation of immortalised human neural progenitor cells (ReNcell VM). Electrical stimulation protocol of 100 Hz DC pulsed stimulation generating a field of 1 V/cm altered the aspect ratio of the cells, producing an elongated cell morphology, relative to unstimulated controls. Furthermore, electrical stimulation significantly increased the percentage of cells positive for Tuj1 (a neural marker β-III tubulin) by 1.6× and enhanced neurite length (108 vs. 73 μm) relative to unstimulated cells. The micropatterning of OCP films has also been demonstrated to provide the addition of favourable topographic cues advantageous to the organisation of neuronal cells. Employing a micropatterning technique, Wu et al. (91) fabricated a conductive poly(glycerol sebacate)—aniline pentamer polymer presenting aligned grooves, with Schwann cell (SC) alignment and elongation enhanced on the conductive micro-grooved scaffolds, as was gene expression for nerve growth factor, relative to micropatterned non-conductive controls.

The modification of the conducting polymer interface with extracellular matrix proteins has been one approach explored to enhance the direct interfacing between neuronal cells and OCP biomaterial surface. Type I collagen, covalently linked to polypyrrole doped with the biological dopant chondroitin sulphate, increased cell proliferation by 42% after 24 h, and 340% after 168 h. Cell differentiation and neurite outgrowth of rat pheochromocytoma (PC12) cells on the collagen modified polymers was also improved on collagen modified PPy, which was further enhanced by electrical stimulation (89). In another study, biological dopants, including those derived from algae, and extracellular matrix components, were employed to improve biological interfacing of PC12 cells with PEDOT biomaterials, relative to PEDOT doped with synthetic chemistries (36). PEDOT doped with the large molecular weight algal polysaccharide Ulvan demonstrated the greatest number of cells after 72 h cell culture relative to all other PEDOT films. Electrical stimulation experiments [±0.25 mA cm^−2^ with biphasic waveform of 100 μs pulse (250 Hz)], comparing PEDOT-Ulvan with PEDOT doped with dodecylbenzenesulfonic acid (DBSA), showed electrical stimulation on both PEDOT films to significantly enhance PC12 differentiation markers (e.g., mean branches per cell, maximum neurite length per cell, mean neurite length per cell, mean neurites per cell and total outgrowth length per cell), relative to unstimulated cells and those grown on tissue culture plastic. When comparing the degree of differentiation vs. non-differentiated cells, the highest rate of differentiation was illustrated on electrically stimulated PEDOT-ULV (97%), followed by electrically stimulated PEDOT-DBSA (96%), non-stimulated PEDOT-ULV (95%), tissue culture plastic (93%) and non-stimulated PEDOT-DBSA (90%).

Electrospinning has been widely explored in the fabrication of conductive and biodegradable nanostructured fibrous biomaterials platforms for tissue engineering, presenting features at a scale that may mimic the native extracellular matrix environment. This approach generally involves the electrospinning of a biocompatible and biodegradable material such as polycaprolactone (PCL) and poly(lactic acid) (PLA), followed by the modification of the nanofibers with OCPs by using chemical (75–78) or vapour phase (74) polymerisation approaches, or by mixing of the biodegradable polymer and the OCP material followed by directly electrospinning the polymer composite (55, 56). Lee et al. fabricated random and aligned nanofiber meshes *via* the electrospinning of poly(lactic-co-glycolic) acid (PLGA), which were subsequently modified with a polypyrrole (PPy) sheath using a chemical polymerisation process (75). The proliferation and differentiation of PC12 cells and hippocampal neurons on electrospun PLGA meshes with and without PPy were shown to be comparable, however, with electrical stimulation (10 mV/cm for 2 h after 24 h culture), PC12 cells on the conductive PPy-PLGA mesh demonstrated a 40–50% increase in neurite length, and 40–90% increase in neurite formation relative to an unstimulated PPy-PLGA control. Cells grown on aligned fibre mesh, compared to the randomly orientated mesh, illustrated longer neurites and more neurite bearing cells. In another study, an *in-situ* oxidative process was used to coat electrospun poly(_L_-lactic acid-co-ϵ-caprolactone) [PLCL/silk fibroin (SF)] mats to form an electroactive PPy/PLCL/SF scaffold for neural tissue engineering (77). The randomly aligned mats were functionalised with different amounts of PPy, with the most highly modified scaffolds demonstrating a conductivity of 1.36 × 10^−4^ S cm^−1^. MTT assays showed Schwann cell proliferation to increase with increasing PPy modification and with electrical stimulation (100 mV cm^−1^ for 1 h per day). Immunofluorescence staining showed electrical stimulation capable of increasing PC12 differentiation even in the absence of exogenously supplied nerve growth factor (NGF), with the most highly modified PPy/PLCL/SF demonstrating 99.67 ± 0.47% differentiated cells, relative to 7.67 ± 1.01% for the PLCL/SF control.

The mixing of the biodegradable polymer and OCP materials prior to electrospinning, forming a conductive composite polymer electrospinning solution, has been developed as an approach to remove the requirement for the added step of OCP modification after formation of the electrospun structure (55, 56). A 70:30 poly(ϵ-caprolactone):gelatin (PG) solution was loaded with either 10 or 15 wt% polyaniline (PANI/PG), and composite electrospun mats were deposited and characterised (56). The 15 wt% polyaniline loading was determined to provide the best overall material properties, based on the fibre and pore size, mechanical properties and conductivity, and used for biocompatibility testing with nerve stem cells (NSCs). NSC proliferation over a 7-day period was shown to be slightly higher on the PG control scaffolds, followed the PANI/PG, with tissue culture plastic being significantly lower, at each of day 3, 5, and 7 time points. It was proposed the relevant higher loading of gelatin in the PG sample provided enhanced capacity for cell adhesion and proliferation relative to the PANI/PG scaffold. Electrical stimulation experiments were performed on NSCs 24 hrs post seeding on the PANI/PG, with a potential density of 100 mV/mm applied for 15, 30 or 60 min. While cells stimulated for 15 and 30 min showed no difference compared to unstimulated samples, electrical stimulation for 1 h demonstrated a significant increase in proliferation and neurite length of NSCs (30.11 ± 1.10 μm relative to unstimulated controls 22 ± 0.97 μm). This research group later used a similar approach to blend PANI and poly(_L_-lactide) (PLL) to fabricate conductive composite electrospun scaffolds (55).

Several different approaches have been used to fabricate conductive hydrogel based scaffolds for neural engineering, based on several hydrogel platform materials, including polyesters (149, 150), PEG (151), protein (94) and cellulose (85). PPy-collagen composite fibres were produced using an interfacial polyelectrolyte complexation (IPC) method, producing long micron-scale fibres (94). The electrical stimulation of human mesenchymal stem cells (hMSCs) using a biphasic waveform (1.2 V with pulse duration 5 ms and frequency of 200 Hz) was shown to enhance hMSC proliferation after 5 days relative to unstimulated controls. Early expression of neuronal marker MAP2 and upregulation of synaptophysin, a membrane protein present in synaptic vesicles, was also observed on the stimulated samples after 5 days. However, after 10 days of culture, hMSC proliferation was greatest on unstimulated controls.

Electroactive aerogels composed of nanoporous cellulose gels coated with PPy nanoparticles were fabricated using supercritical CO_2_ drying process, presenting materials with good mechanical and electrical properties (conductivity of 0.08 S cm^−1^) (81). Gels incorporating the PPy nanoparticles demonstrated increased PC12 cell adhesion and proliferation. PC12 neurite extension was shown to be greater on gels with PPy, in particular with PPy polymerised with dodecylbenzenesulfonic acid (DBSA) used as the dopant anion. In another study, chitosan/gelatin hydrogels formed using a crosslinking and freeze-drying approach were coated with PEDOT nanoparticles using an interfacial polymerisation method (58). Modification with PEDOT nanoparticles increased the electrical conductivity, hydrophilicity, thermal stability and mechanical properties relative to the unmodified control hydrogels, while water absorption and biodegradability decreased. The PEDOT modified chitosan/gelatin hydrogel materials promoted the adhesion and proliferation of PC12 cells (90.31 ± 2.11% vs. 80.22 ± 2.11% for the control), as well as PC12 cellular neurite growth, and results were further supported through higher protein and gene expression levels. In a subsequent study from this group, PEDOT was incorporated into a preformed carboxymethyl chitosan (CMC) hydrogel by soaking the gel in an aqueous solution containing the oxidant ammonium persulphate and anion sodium *p*-toluene-sulfonate (pTS) under vacuum, followed by the incubation of the gel in an EDOT/hexane solution, forming the composite PEDOT/CMC conductive hydrogel (85). Optimal conditions were shown to produce PEDOT/CMC hydrogels presenting 1825 ± 135 wt% water, compressive modulus of 9.59 ± 0.49 kPa, porosity of 93.95 ± 1.03% and conductivity of 4.68 ± 0.28 × 10^−3^ S cm^−1^, and shown to be cytocompatible, supporting the adhesion and proliferation of PC12 cells.

**Figure 3:**
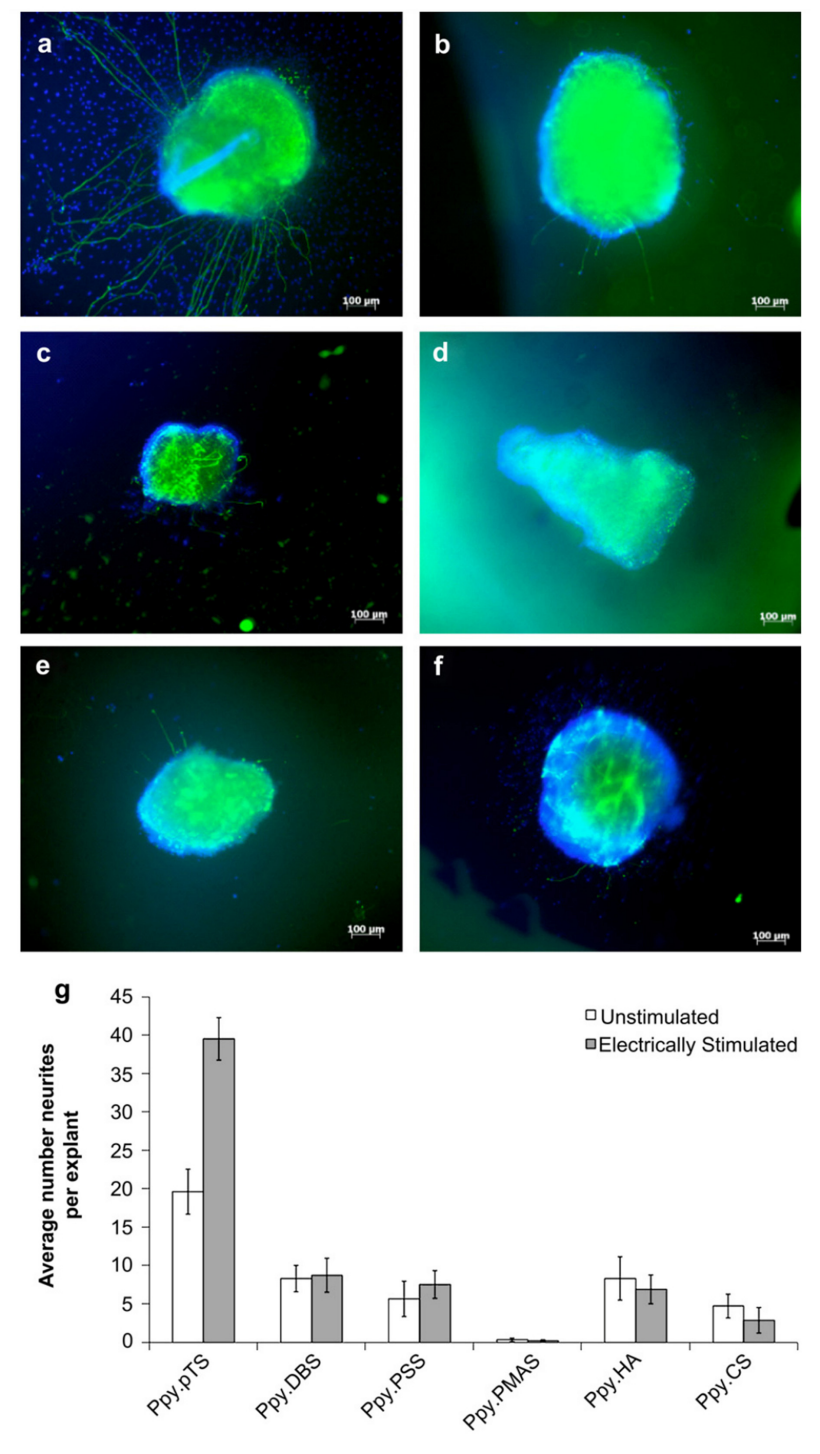
Response of auditory nerve tissue to electrically-controlled NT-3 release from PPy films grown with different dopants. All polymers were grown with 2 mg/mL NT-3 in the polymerisation solution, and 7.2 C of charge were passed in the polymerisation of all films. **(a–f)** Show representative images of explants growing on various PPy films after electrically stimulated release of NT-3 from the PPy. **(a)** Explants on PPy/pTS, **(b)** PPy/DBS, **(c)** PPy/PSS, **(d)** PPy/PMAS **(e)** PPy/HA and **(f)** PPy/CS. **(g)** The average number of neurites extending from explants grown on electrically-stimulated and unstimulated films over six experiments. The error bars show one standard error of the mean of 6–60 explants on each polymer. Reproduced with permission from Thompson et al. (64).

The ability for OCPs to undergo redox processes, including the release of the incorporated ions during electrochemical polymerisation, has been exploited for application of these materials as controlled drug delivery devices, and have been explored for use in neural tissue engineering (64). Thompson et al. (64) electrochemically polymerised PPy films with several synthetic and biological dopant species, determining their biocompatibility by measuring their capacity to support neurite extension from spiral neural ganglion (SNG) explants. PPy films polymerised with the dopant DBSA illustrated the greatest degree of neurite extension from the explants (>50 neurites per explant vs. <33 for the next closest sample PPy-pTS). Thereafter, the neural growth factor neurotrophin-3 (NT-3) was incorporated into the polymer films as a co-dopant, and its release profile and biocompatibility were further determined with and without electrical stimulation. PPy-pTS co-doped with NT-3 showed the greatest NT-3 release for electrically stimulated films over a 7-day period (8 ng/cm^2^ per day), and the greatest number of neurites per explant (~20 neurites per explant) among all NT-3 co-doped PPy films, while electrical stimulation producing a significant increase in the number of neurites relative to the unstimulated film (~40 neurites/explant) (Figure 3). A different approach was taken by Gomez and Schmidt (90), whom rather than incorporating the neurotrophin as the dopant, chose to immobilise it on the surface of electrochemically polymerised PPy-PSS films. Nerve growth factor (NGF) immobilised at surface densities of 0.21–1 ng mm^−2^ resulted in an increase in neurite producing cells, relative to no NGF, and was comparable to delivery of NGF in solution (50 ng/ml), after 2 days in culture. However, while PPy-PSS films with the highest density of immobilised NGF presented comparable numbers of neurite producing cells after 10 days culture, all lower NGF immobilisation densities demonstrated a significant reduction relative to 2-day values. Electrical stimulation of PC12 cells (100 mV applied for 2 h, 24 h prior to characterisation) showed an enhancement in neurite length for PPy-PSS-NGF films (median length of 12 μm), relative to unstimulated controls (median length of 8 μm), however PPy-PSS controls with NGF in solution still presented cells with greater neurite length, thus presenting possible limitations in the application of NGF immobilisation on the polymer surface as an effective approach to enhancing neural tissue differentiation. Drug loaded conductive hydrogels have also been explored for neural tissue applications, with the reaction of an aniline dimer and gelatin used to form an electroactive hydrogel biomaterial, which was loaded with the model drug dexamethasone (93). The conductive hydrogels were shown to present good mechanical and electrical properties, and illustrated an increased drug release as a function of electrical stimulation. PC12 cell viability, adhesion and neurite extension were best on the 10 wt% aniline hydrogels, presenting this as a promising system for further tissue engineering applications.

Several studies have illustrated the efficacy of OCP based electroactive conduits for neural tissue engineering *in-vivo* (77, 95, 152, 153). Abidian et al. (95) fabricated a reinforced agarose nerve conduit made conductive through the partial or full coating of the lumen by a thin layer of electrodeposited PEDOT. Control and conductive conduits were implanted in a 10 mm peroneal nerve gap in a rat model, with the efficacy for the conduit to reinnervate this gap determined using extensor digitorum longus (EDL) muscle contractile force measurements. EDL measurements were shown to be the poorest for the unmodified hydrogel conduit (26 ± 4 mg), with a significant increase in EDL force measured for the two PEDOT modified conduits (60 ± 11 and 54 ± 6 mg), while the autograph control performing the best of all treatments (107 ± 30 mg). This demonstrated the ability for PEDOT to enhance neural regeneration *in-vivo*. Another study fabricated a conductive nanofibrous nerve guidance conduit through the electrospinning of a poly(l-lactic acid-*co*-ε-caprolactone)/silk fibroin (PLCL/SF) solution onto a tubular scaffold, followed by modification with PPy-pTS by chemical polymerisation (92). The electroactive nerve guidance conduit was used to repair a 10 mm sciatic nerve gap in a rat model, and characterised at either 4- or 12-weeks post implantation. Histological analyses revealed during the initial period post-surgery (0–4 week), PPy coated conduits could promote Schwann cell proliferation, while myelin formation was enhanced in the later stages post-surgery (4–12 weeks). Nerve regeneration for the PPy coated conduit was similar to the autograph control group, and better than the unmodified PLCL/SF group.

### Cardiac Tissue

Electrical signal propagation through cardiac cells and tissues to facilitate synchronised contraction of the heart muscle, and maintenance of heart function and rhythmic beating, is critical to the proper functioning and maintenance of cardiac tissue. The role of electrically driven processes in the tissue highlights the clear potential benefits of applying electrically conductive materials for therapeutic and tissue engineering for cardiac tissue. OCP films have been shown to support the adhesion and proliferation of cells from a range of cardiac cell models (96, 97, 154, 155). PANI films, both in conductive and non-conductive forms, were shown to illustrate slightly reduced cell adhesion of H9c2 cardiac myoblasts (<7%) relative to tissue culture plastic, however cell proliferation rates were comparable over a 6 day culture period, demonstrating good cytocompatibility (96). In another study, a flexible film composed of PANI doped with phytic acid on a chitosan film was develop as an electroactive and flexible material for cardiac models that can critically maintain conductivity over an extended time period (97). Therein the authors demonstrated the strong chelation between the phytic acid and chitosan led to the retention of the anion within the scaffold, allowing the patch to retain good electroactivity, low surface resistivity and an oxidised form after 2 weeks incubation in physiological media. Application of the film in *ex-vivo* heart models demonstrated the patch to have a favourable influence on the electrophysiology of the tissue, which, coupled with the excellent stability of OCP films properties, presented a good system for further investigation. A conductive interpenetrating network of PPy in a flexible PCL film was used to form a biomaterial platform presenting conductivity similar to native cardiac tissue (resistivity of 1.0 ± 0.4 k Ohms cm^−1^) (98). Cardiac myocytes cultured on the PPy/PCL materials were shown to have a greater peripheral localisation of the gap junction protein connexin 43 (Cx43), compared to cells on PCL alone, however the gene expression of connexin 43 was unchanged between the two materials. Critically, the authors observed that calcium wave propagation was faster and calcium transient duration shorter for cardiomyocyte monolayers on the PPy/PCL (1,612 ± 143 μm/s, 910 ± 63 ms) relative to cells on PCL (1,129 ± 247 μm/s, 1,130 ± 20 ms), respectively.

The electrospinning of conductive mats has been an attractive approach for cardiac based applications, providing freestanding, porous and flexible films that can be applied directly onto dynamic cardiac tissue *in-vivo* (71, 98–100, 156). Electrospun fibres composed of PPy/PCL/gelatin were fabricated using different percentage loadings of PPy (15 or 30 wt%) (71). Composites composed of 15 wt% PPy presented the most favourable electrical, mechanical and biodegradable properties, with cell proliferation also the greatest over 8 days of cell culture, relative to 30 wt% PPy/PCL/gelatin and PCL/gelatin scaffolds. Furthermore, the 15 wt% PPy scaffolds showed a high expression of the cardiac specific proteins α-actinin, troponin-T and Cx43, presenting their potential use in cardiac tissue engineering. Hsiao et al. (99) developed a mesh containing aligned composite nanofibers of PANI and PLGA as an electrically active scaffold capable of co-ordinating the synchronous beating of cultured cardiomyocytes. Protonation (or doping) of the PANI using HCl was shown to enhance the adsorption of the extracellular matrix proteins laminin and fibronectin, and promote cell adhesion. Cells within each cell cluster on the doped PANI/PLGA fibres demonstrated synchronous beating, although separate cell clusters on the same mesh beat asynchronously. Application of an electrical stimulus (1.25 Hz, 5 V/cm) resulted in the synchronous beating of all cell clusters at the same frequency as the electrical stimulus. In another study, electrospun PLA sheets containing 1 and 3% PANI were shown to enhance H9c2 cardiomyocyte differentiation, relative to electrospun PLA controls (100). Incorporation of PANI into the electrospun sheets also increased the expression of both α-actinin and Cx43, relative to the PLA control. Functional and biological behaviour of the cells on the materials were investigated by looking at the maturation and beating behaviour. Cells grown on the PANI incorporating sheets showed a more synchronous beating pattern with a higher beat rate than on the PLA sheets alone as early as 4 days. This was thought to result from the PANI/PLA sheets with conductivity promoting the formation of electrical coupling of cells during spontaneous beating, thus establishing a more synchronous beating pattern.

Conductive hydrogels have also shown promise for cardiac tissue engineering applications. A PPy-chitosan hydrogel (3:10 ratio) was formed through a chemical oxidative polymerisation method, and illustrated comparable cell adhesion and proliferation of smooth muscle cells to chitosan controls, while neonatal rat cardiomyocytes plated on the PPy/chitosan gels showed improved Ca^2+^ signal conduction relative to chitosan alone (86). Injection of the PPy-chitosan hydrogel into a rat model 1 week after a myocardial infarction improved electrical function, relative to the injection of a saline or chitosan control. In another study, Dong et al. (43) developed an injectable, conductive and self-healing hydrogel based on a chitosan-*graft*-aniline tetramer and dibenzaldehyde-terminated poly(ethylene glycol), as a cell delivery vehicle for treatment of myocardial infarction. The porous hydrogel possessed a similar conductivity to native myocardium (2.29–2.42 × 10^−3^ S cm^−1^), was demonstrated to adhere well to host tissue, and showed good cell viability and proliferation of encapsulated C2C12 and H9c2 cells.

### Skin Tissue Engineering

Several approaches have been used to fabricate electroactive OCP films for skin tissue engineering, including electrochemical (101, 102), chemical (66) and vapour phase (53) polymerisation, and the blending of OCP nanoparticles with biodegradable polymers (67, 68, 104, 114, 157). Amine functionalisation of electrochemically polymerised PPy/pTS films were shown to provide a beneficial surface for the adhesion of Schwann cells and fibroblasts, owing to the charge – charge interactions between the modified polymer film and the cell membrane, relative to unmodified films (101). Lee et al. (102) electrochemically polymerised carboxylic acid functionalised terthiophene films that presented carboxylic functional groups to allow the covalent modification of the films with cell adhesion peptides (i.e., RGD). The polymers showed good conductivity (1.22 ± 0.15 S cm^−1^), with studies using human dermal fibroblasts showing the RGD modified films to show improved cell adhesion by ~200%, relative to unmodified controls, after 4 h. In another study, a pulsed electrical field stimulation was used to stimulate human primary skin fibroblasts on a PET fabric modified with PPy using a chemical polymerisation method (66). The stimulation protocol (100 mV mm^−1^) produced an upregulation of TGFβ1, which promoted the transdifferetiation of fibroblast to myofibroblast throuth the TGFβ1/ERK/NF-κB signalling pathway. Furthermore, the electrical stimulation activated fibroblast phenotype change was retained after prolonged culture, indicated by the gene expression and immunocytochemistry staining of α smooth muscle actin.

**Figure 4:**
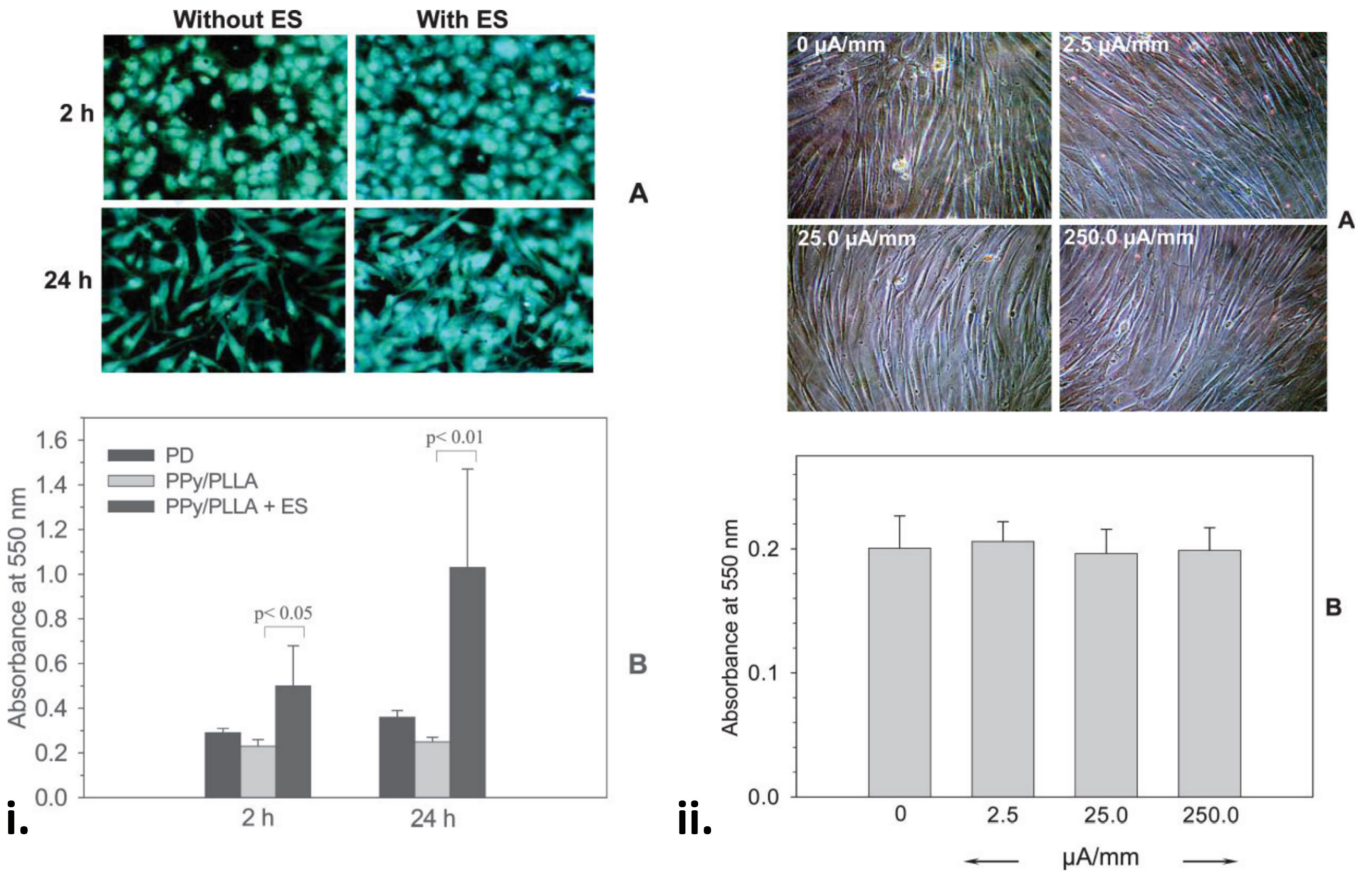
(i) Fibroblasts on the PPy/PLLA membranes at 2 and 24 h with or without ES. Note the comparable cell distribution and high cell density on the ES membranes **(A)**. **(B)** Shows a significantly higher cell viability on the membranes with ES (100 mV/mm). (ii) Fibroblasts on the gold-coated tissue culture Petri dish for 96 h with or without ES at various surface current densities, showing that a wide range of DC current density had no effect on cell morphology **(A)** and viability **(B)**. Reproduced with permission from Shi et al. (103).

The blending of OCP nanoparticles with biodegradable polymers has been widely used to fabricate electroactive and degradable composites for wound healing applications (67, 68, 103, 104, 157–159). Composite films containing 5% PPy nanoparticles with 95% biodegradable poly(_L_-lactide) (PLLA) were fabricated and tested against the adhesion of human cutaneous fibroblasts in the presence or absence of an applied direct electrical current (DC) ranging from 50 to 500 mV/mm (67). The PPy/PLLA films were shown to support cell adhesion, spreading and proliferation in the presence and absence of electrical field lower than 100 mV/mm, however, electrical stimulation dramatically increased cytokine secretion by ~10-fold compared to non-stimulated controls. In another study from this group (103), PPy/PLLA composite membranes were fabricated using drop casting and tested for their ability to support the culture of human cutaneous fibroblasts with and without electrical stimulation. The application of a 100 mv/mm electrical field improved cell viability on the PPy/PLLA membranes at 2 and 24 h by 2.2- and 4.0-fold, respectively, demonstrating the ability for the application of electric fields on the PPy/PLLA materials to improve human skin fibroblast viability and culture (Figure 4). The incorporation of heparin into PPy nanoparticles has also been shown to improve cell adhesion and growth of human dermal fibroblast in PPy-PLLA composite films (104).

Both aligned and randomly distributed electrospun conductive scaffolds have presented good properties for fibroblast adhesion and growth (72, 73, 105, 160–163). PANI doped with camphorsulfonic acid (CPSA), and blended with poly(_L_-lactide-co-ϵ-caprolactone) (PLCL), was electrospun to prepare randomly aligned nanofibre mats (105). PANI/CPSA was incorporated to differing degrees (0–30 wt%), with fibre diameters ranging from 100–700 nm. The adhesion of human dermal fibroblasts and NIH-3T3 fibroblasts was significantly higher on the PANI/CPSA/PLCL scaffolds, relative to pure PLCL, and growth of NIH-3T3 fibroblasts was enhanced as a function of direct current electrical stimulation. Random and aligned electrospun mats of a PANI-chitin composite were also both shown to support the adhesion and viability of human dermal fibroblasts, although cell polarisation was enhanced on the aligned scaffolds, as was cell viability which was ~2.1-fold higher relative to random scaffolds after 7 days of culture (72). In another study, PANI-chitosan electrospun fibre scaffolds showed that increasing the PANI content in the composite blend (PANI-chitosan ratio of 1:3, 3:5 or 1:1) resulted in higher hydrophobicity and conductivity of the materials, and supported the attachment and growth of osteoblast and fibroblast cells (73). The PANI-chitosan ratio of 1:3 was shown to present the best attachment and proliferation for both cell types.

### Skeletal Muscle Tissue Engineering

OCP and OCP-composite biomaterial films have been demonstrated to support the adhesion, proliferation and differentiation of skeletal myoblasts (27, 69, 107, 108, 164). Gilmore et al. showed that PPy films doped with biological or synthetic dopants were able to support the proliferation and differentiation of C2C12 myoblast cells, although some variations in both proliferation and differentiation were reported as a function of the different dopants (27). A flexible and degradable electroactive shape memory polymer based on an amino capped aniline trimer was also shown to enhance proliferation and differentiation of C2C12 cells, with myosin heavy chain (MHC) and myogenin gene expression increased by up to 2× relative to pure PCL control films (107). The elastic properties of polyurethane, providing beneficial mechanical properties for skeletal muscle cell and tissue interfacing, has seen PPy-polyurethane composite polymers used to provide conductive polymeric films that present good cytocompatibility (69), while an electroactive polyurethane – urea co-polymer, synthesised with an amine capped aniline trimer, showed good cell viability relative to polyurethane and PLLA controls, and demonstrated increased myotube density, myotube length and myotube maturation index values relative to PLLA films, presenting the conductive composite films as attractive substrates for myogenic tissue engineering (108).

Electrospinning has been applied to form highly conductive composite nanofibre scaffolds for skeletal muscle engineering based on both random and aligned nanofiber morphologies (20, 109, 110, 165–167). The electrospinning of poly(_L_-lactide-co-ϵ-caprolactone) (PLCL) blended with PANI (PLCL/PANI) were shown to present comparable fibre diameters and wettability, regardless of the degree of incorporation of PANI, relative to PLCL control fibres (109). After 4 days, the number of cells positive for sarcomeric myosin was 3.6 times greater on the electrically conductive fibres incorporating PANI, than on the PCL control, and the expression of myogenin, tropionin T and myosin heavy chain (MHC) genes were also enhanced (1.6, 2, and 3-fold, respectively) on the conductive, compared to non-conductive, scaffolds.

The alignment of myoblasts on biomaterials has been demonstrated to improve myogenic activity of biomaterials, and therefore several studies have acted to use electrospun aligned nanofibrous mats to provide suitable topographical cues for the alignment of myoblasts and myotubes (20, 110, 165, 166). Scaffold mats of aligned and randomly orientated nanofibers of PANI/PCL were produced using 3 wt% loading of PANI (110). The PANI/PCL scaffolds showed good conductivity (63.6 ± 6.6 mS cm^−1^), with aligned scaffolds of both the PCL control and PANI/PCL able to orientate the adhered myoblast cells, and promote an increase in myotube formation (~40 and 80%, respectively), relative to the randomly orientated PCL control scaffolds. Aligned PANI/PCL materials showed enhanced myotube maturation relative to aligned PCL or random PANI/PCL materials, demonstrating both conductivity and alignment to provide cues that can enhance skeletal muscle regeneration.

**Figure 5:**
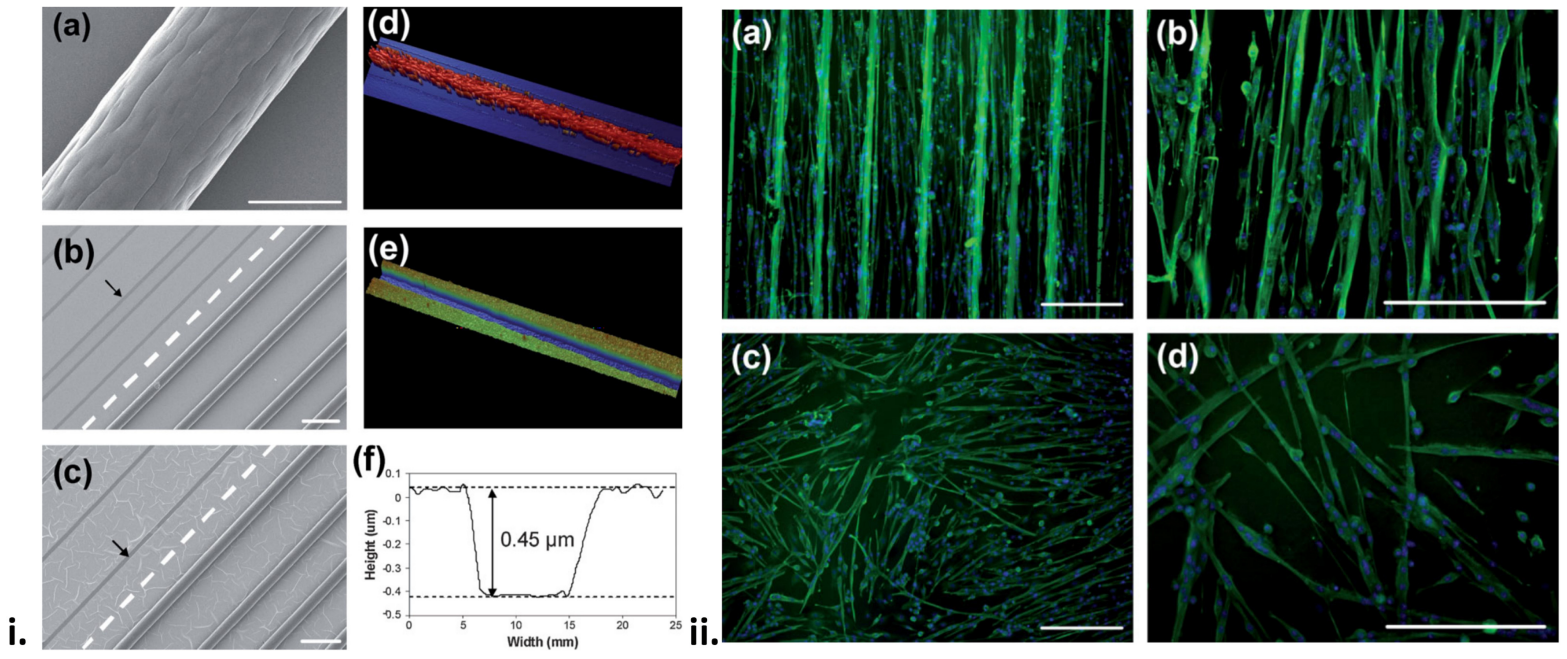
(i) Surface profile of the biosynthetic cell culture platform. Scanning electron microscopy (SEM) images of **(a)** a 75:25 PLA:PLGA fibre, and **(b,c)** hybrid platforms made from 75:25 PLA:PLGA fibres and PPy doped with pTS **(b)** and HA **(c)**. The broken line separates the area on the PPy substrate where the fibres have been removed (pointed by the arrows). Also shown are profilometry images of the hybrid platform in the **(d)** presence and **(e)** absence of PLA:PLGA fibres, and **(f)** height profile obtained from image **(e)**. (ii) Fluorescence images of differentiated, multinucleated desmin (green) expressing myotubes on PPy/pTS substrate **(a,b)** with and **(c,d)** without the presence of PLA:PLGA fibre array. Cell nuclei are shown in blue. Scale bars are 200 mm. Reproduced with permission from Razal et al. (168).

Alternative approaches have been used to generate OCP materials that present topographic cues aimed at aligning myoblast cells on the materials. Aligned multiwall carbon nanotube (MWCNT) mats were used as a platform film on which PPy-pTS was electrochemically polymerised (106). MWCNT-PPy-pTS materials were shown to promote the alignment of ROSA primary skeletal myoblasts, compared to PPy-pTS films alone. Electrical stimulation of cells on the MWCNT-PPy-pTS films (0.125 mA cm^−2^ bipolar square wave with 2 ms pulses at 10 Hz) increased total cell nuclei, and myoblast fusion, compared to unstimulated films. In another study (168), aligned wetspun fibres of PLA:PLGA were deposited onto a PPy film doped with either chondroitin sulphate, pTS, hyaluronic acid, or poly(3-methoxyaniline-5-sulfonate) (PMAS). The presence of the aligned PLA:PLGA fibres was shown to direct the alignment, growth and differentiation of attached ROSA primary skeletal myoblasts, with the PPy-PMAS films with the PLA-PLGA fibres shown to generate the most pronounced myodifferentiation of all scaffolds (Figure 5).

### Bone Tissue Engineering

OCP PPy/heparin (PPy/He) nanoparticles mixed with PLLA (5 wt% PPy/He), and cast to form a PPy/PLLA conductive membrane, have been shown to support osteoblast adhesion and bone mineralisation processes (114). Osteoblast-like Saos-2 cells seeded onto the conductive membranes were stimulated with a constant potential of 100, 200, 300 or 400 mV/mm for periods ranging from 2 to 8 h at each intensity. Forty-eight hours after stimulation, the 200 mV/mm stimulation protocol was shown to significantly increase osteocalcin gene activation and protein secretion. Osteocalcin is an osteo-blast secreted, non-collagenous protein present in bone and dentin, and plays a crucial role in bone mineralisation. In another study, the attachment and proliferation of bone marrow stromal cells (BMSCs) and MC3T3-E1 (pre-osteoblast) cells to self-doped sulfonated PANI interdigitated electrodes was shown to increase with increasing sulfonation of the PANI films (112). Alkaline phosphatase (ALP) activity of the cells under electrical stimulation (1 kHz at 500 mV) showed MC3T3-E1 cells to illustrate an increase in ALP activity at days 6, 9, and 12, relative to unstimulated controls. Electrical stimulated samples also presented greater mineralisation relative to non-stimulated samples.

**Figure 6:**
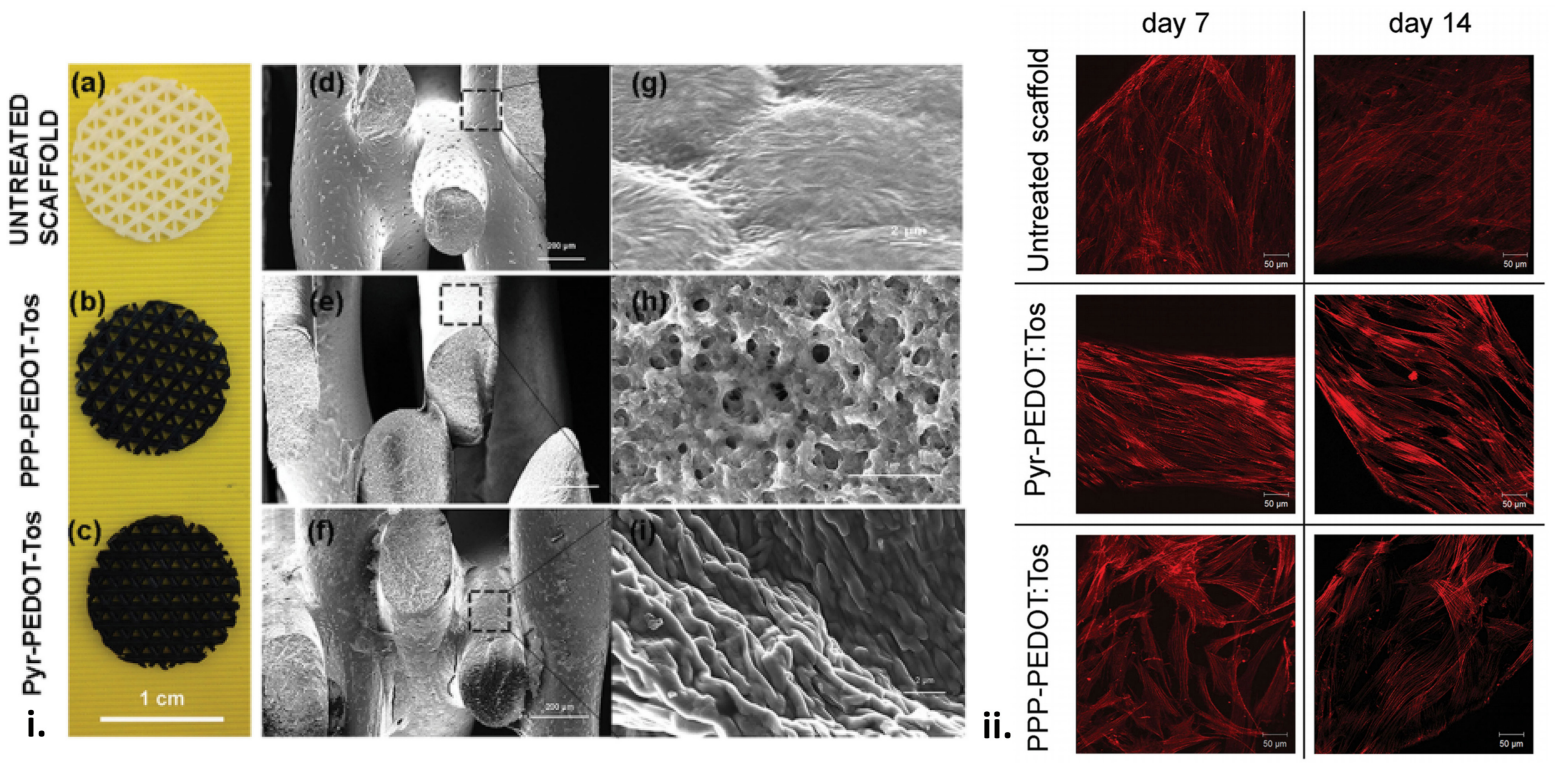
(i) Scaffolds surface structure. **(a,d,g)** Pictures of the uncoated and coated scaffolds and SEM images of the macroporous scaffolds before and after VPP using the two different oxidant solutions. **(b,e,h)** PPP-PEDOT:Tos scaffolds and **(c,f,i)** Pyr-PEDOT:Tos. **(a–c)** scale bar: 1 cm, **(d–f)** scale bar: 200 μm, **(g–i)** scale bar: 2 μm). (ii) F-actin staining of foetal mesenchymal stem cells seeded on untreated and coated scaffolds. Z-stack images were taken for cells after 7 and 14 d of proliferation on different scaffolds surfaces. Scale bar: 50 μm. Reproduced with permission from Iandolo et al. (70).

Several studies have developed PLA – OCP composites incorporating PANI (113, 115, 169) and PEDOT (70), demonstrating their efficacy at promoting osteogenesis with a range of cell types. An *in-situ*, thermal induced phase separation (TIPS) method was used to fabricate conductive nanofibrous PLA scaffolds with well-distributed PANI nanostructures (115). When tested against bone marrow derived mesenchymal stem cells, expression levels of ALP, osteocalcin and runt-related transcription factor 2 (RUNX2) were all increased with only a moderate level of PANI (10%), relative to PLA alone, indicating the osteogenic potential of the scaffolds. A shape memory polymer fabricated using star shaped PLA and an aniline trimer was shown to exhibit excellent mechanical properties (modulus in the GPa range) and excellent shape memory performance (113). The polymer containing aniline demonstrated increased proliferation, and differentiation of C2C12 cells towards an osteogenic lineage, based on ALP enzyme activity and immunofluorescence staining of RUNX2 protein, and gene expression assays for ALP, RUNX2, and type I collagen. In another study, macroporous PCL scaffolds were modified with PEDOT using a vapour-phase polymerisation process, providing added electronic functionality to the osteogenic scaffolds (Figure 6) (70). Therein the cytocompatibility of the PCL and PEDOT/PCL scaffolds, tested using mesenchymal stem cells, were shown to be comparable, indicating their further potential for osteoinduction and therapeutic applications.

Several studies have also developed OCP based composite scaffolds using inorganic components, including titanium nanotubes (111), bioglass (170) and mesoporous silica nanparticles (116). Titanium nanotubes (TNTs) coated with PANI *via* a potentiostatic polymerisation process were shown to present large surface area to volume scaffolds where the PANI coating improved biocompatibility, electrical conductivity, hydrophilicity and biomineralization (111). The PANI coated TNTs possessed antibacterial properties, and osteogenic potential through increased proliferation of Mc3T3-E1, and ALP activity, relative to the TNT control. Collagen I secretion, a major indicator for osteoblastic phenotypes, was also greatest on the PANI-TNT materials, relative to the TNT and titanium controls, indicating an increase in osteoblastic differentiation, and thus presenting more mature cells after the given period of culture. In another study, porous and conductive scaffolds were generated by mixing hydroxyapatite, gelatine, mesoporous silica and PPy, and formed using a freeze drying method (116). Loaded with the antibiotic vancomycin (VMC), scaffolds containing PPy showed good mechanical properties, conductivity, higher levels of protein adsorption and higher VCM release over a 120-day period. They were shown to support the growth of osteoblast cells, and promoted the formation of calcium phosphate deposits quicker than control scaffolds not containing PPy.

## Graphene

### Neural Tissue Engineering

The first studies to demonstrate the efficacy of graphene as a biomaterial were undertaken using pristine graphene films fabricated *via* chemical vapour deposition (CVD). The application of graphene for neural tissue engineering were a significant focus of these early investigations, with CVD grown graphene on polyethylene terephthalate (PET) shown to enhance cell-cell interactions of SHSY5Y human neuroblastoma cells using electrical field stimulation (45). CVD grown graphene was also shown to significantly support the number of differentiated human neural stem cells on graphene (225 ± 13 cells per area) compared to glass (114 ± 13 cells per area) after prolonged culture (1 month) (46), as well as promote the conversion of mesenchymal stem cells towards a neuronal lineage (61), with the latter study showing cells grown on graphene substrates (relative to a glass control) to exhibit significantly higher expression of neurogenesis related genes nestin (early marker), Tuj1 (early marker) and MAP2 (later marker), even in the absence of neurogenic inducers.

The CVD fabrication approach has been applied not only to fabricate 2D films, but also 3D porous scaffolds, producing structures that allow cells to grow into more complex 3D morphologies, and that more closely represent native biological structures. A 3D porous nickel template was used to produce CVD graphene foam (44). Microglia, a type of glial cell known to mediate inflammatory responses in the central nervous system, were grown on the 2D graphene films and 3D graphene forms, respectively, and the supernatant was collected after 24 h and used as conditioned medium for the culture of neural stem cells. It showed that different culture conditions (TCPs, 2D and 3D graphene structures, respectively) can induce different level and type of secretion from microglia and therefore influence NSC behaviour. Conditioned medium taken from microglia grown on the 3D graphene foams demonstrated NSC behaviour far superior to that from the TCPs and 2D films, promoting neurosphere formation and NSC migration, as well as increasing single cell polarisation and cell adhesion to the substrate.

**Figure 7:**
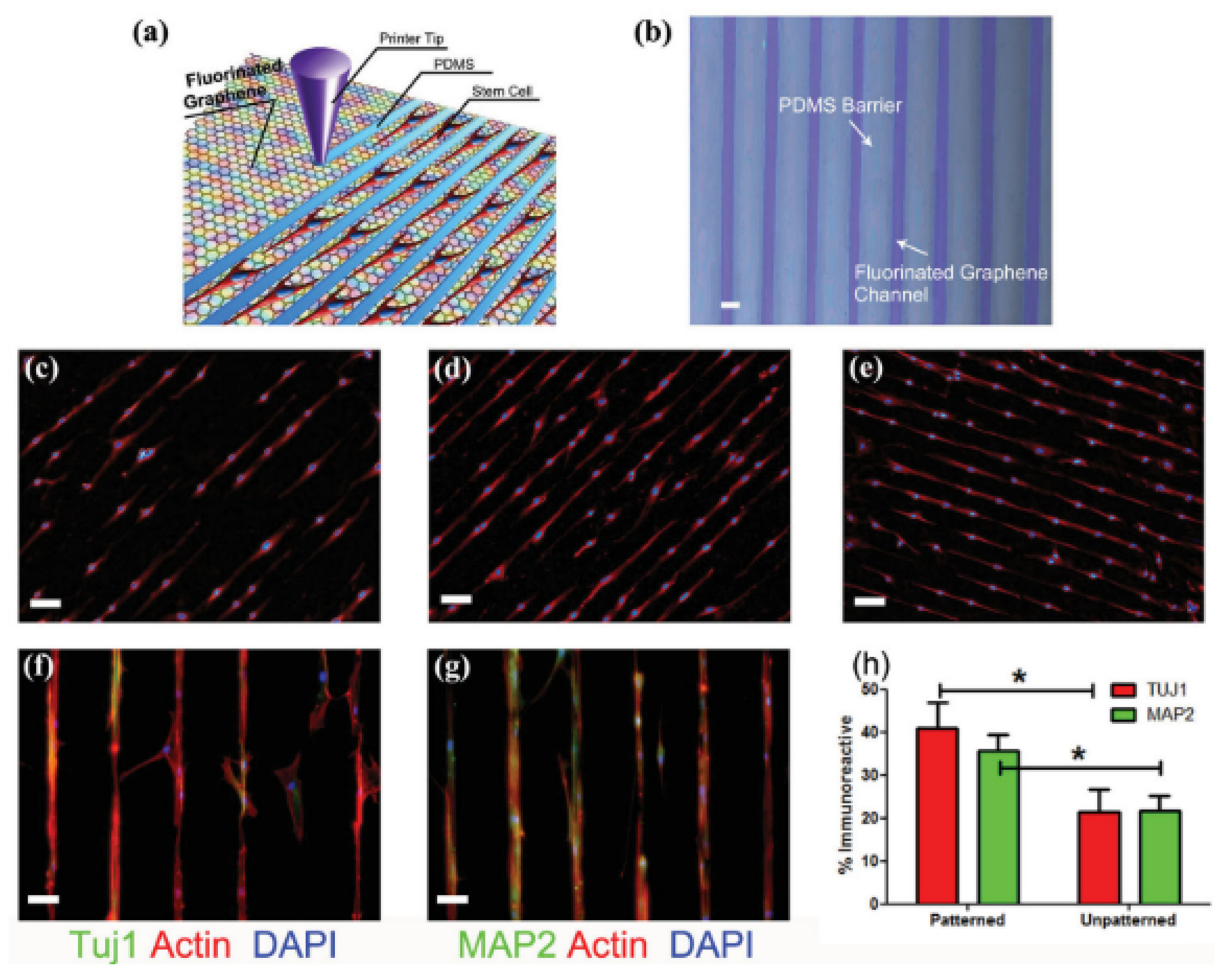
**(a)** Schematic drawing of patterning MSCs by printing PDMS barriers on graphene films directly. **(b)** Optical microscope image of printed PDMS on fluorinated graphene film (scale bar = 50 μ m). **(c–e)** The aligned growth of stem cell on graphene, PFG and FG with printed PDMS pattern, respectively (scale bar = 100 μm). **(f,g)** MSCs preferentially attached on the FG strips and their F-actin aligned (red) and expressed neural specific markers- Tuj1 and MAP2 (green) (scale bar = 50 μ m). **(h)** Percentage of immunoreactive cells for Tuj1 and MAP2 on unpatterned and patterned FG strips. Note that the patterned FG strips induce higher expression of Tuj1 and MAP2 in the absence of retinoic acid (*n* = 6, *p* < 0.05). Reproduced with permission from Wang et al. (117). *signifies statistically significant differences between samples.

The incorporation of surface topographical features on graphene-based biomaterials has proven to be a beneficial approach to guiding neuronal cell behaviours on biomaterial surfaces (117, 118, 171). Wang et al. used fluorinated graphene sheets as a platform for mesenchymal stem cell growth, with the incorporation of PDMS channels used to guide cell alignment to enhance neurogenesis (117). Therein they showed the incorporation of 30 μm PDMS microchannels enhanced gene expression of Tuj1 and MAP2 above surfaces without channels, even in the absence of a neural inductive agent (Figure 7). A graphene-chitosan composite was shown to promote the adhesion and differentiation of human mesenchymal stem cells above that for chitosan films alone, with the underlying mechanism of enhanced initial adhesion, cell-substrate interactions and cell – cell contacts proposed to result from nanoscale topographic cues on the graphene-chitosan materials (118). This resulted in the degree of neurogenesis (number of cells stained with Tuj1 and NeuN/total number of cells) being 2× greater on 5% graphene-chitosan materials relative to the chitosan control. In another study, graphene oxide (GO) was deposited on 300 nm silica nanoparticle (SiNP) to form a nanostructured GO substrate (171). Neuronal differentiation and axonal alignment of NSCs was enhanced on GO and SiNP-GO surfaces, relative to glass and SiNP controls, with Tuj1, MAP2 and synapsin expression greater on the SiNP-GO surface, relative to GO and SiNP alone.

The 3D fabrication of graphene and graphene-composite scaffolds has evolved to include the production of materials with bulk porous properties such as aerogels and foams (119, 172), as well as intricately designed structures and architectures produced *via* electrospinning (47, 48), wetspinning (49), moulding (50, 122) and 3D printing (123) approaches. A composite scaffold of reduced graphene oxide (rGO) and type I collagen was fabricated by depositing a single layer of rGO sheets onto a porous type I collagen template, producing a biodegradable, conductive and biocompatible scaffold (119). Gene expression and protein characterisation showed that culturing of MSCs on collagen scaffolds with, as opposed to without, the rGO enhanced the differentiation of MSCs towards the neuronal lineage after 7 days culture under neural differentiation conditions, with nestin, Tuj1 and MAP2 expression in the differentiated cells on the rGO modified scaffolds increasing by 2.597 ± 0.422-fold, 5.987 ± 0.826-fold and 15.321 ± 0.804-fold, respectively. In a series of papers, López-Dolado et al. (120, 121) demonstrated the *in-vivo* efficacy of porous scaffolds composed of rGO, fabricated using an ice segregation-induced self-assembly method. These materials were deemed good candidates for this application not because of their electrical properties (with conductivity particularly low – 10^−3^ S cm^−1^), but because of their excellent mechanical properties, with compression studies showing excellent flexibility in both longitudinal and transversal directions. rGO scaffolds were implanted into a rat model following hemisection of the spinal cord, with no local or systemic toxic responses evident (121), and animals implanted with the rGO containing scaffold showing better injury stabilisation and sealing than those without the scaffold (120). Furthermore, GFAP+ cells and pro-regenerative macrophages were seen to be present at the scaffold interface, with the scaffolds also shown to promote angiogenesis within the scaffold (120).

Nanoporous electrospun scaffolds have been produced either by incorporating graphene into the spinning solution (47), or depositing of graphene onto a prefabricated electrospun scaffold (48). Graphene-PVA:alginate mats containing 0–5 wt% graphene were shown to produce strong and conductive nanofibrous scaffolds suitable for neural tissue engineering (47). 1 wt% scaffolds showed a 4-fold increase in toughness, as well as enhanced electrical properties (impedance ~25 Ohms). PC12 cell survivability was best on 1 wt% graphene scaffolds after 7 days cell culture. Feng et al. modified a prefabricated electrospun scaffold of poly(vinyl chloride) with graphene oxide nanosheets, reducing the GO after deposition onto the nanofibres (48). Neurons cultured on the scaffolds with deposited GO exhibited increased neurite proliferation and more neurite branches than on TCP and graphene film controls, with neurons also demonstrating a greater level of maturation, as measured by expression of tau protein (a neuraxon marker protein) (i.e., 75% of neurons expressed tau protein on the graphene modified nanofibers vs. 45% on GO film and tissue culture plastic controls).

The application of micron-scale fibres has also been shown as an excellent approach to guide and enhance neural cell adhesion, migration and differentiation. Bendable and conductive GO microfibres were fabricated using a moulding technique, producing fibres of ~50–130 μm in diameter (50). Fibres coated in the neural adhesive molecules N-cadherin and poly(_L_-lysine) were shown to be supportive substrates for the development of interconnected cultures of neurons and glial cells for up to 21 days. *In-vivo* studies showed the implanted microfibres to provide a good guidance platform for an injured rat spinal cord, without obvious signs of subacute local toxicity. In another study, an integrated moulding technique was used to build up a multilayered tubular structure that was composed of four concentric circles composed of a GO-polycaprolactone (PCL) composite (122). GO/PCL composites containing 1% GO presented optimal cell proliferation for all composites, and presented similar values to TCP and PCL controls. The expression of several proteins and genes important for cell proliferation, adhesion, ECM formation and neuronal markers (i.e., Ki67, N-cadherin, vinculin, integrin, Tuj1, nestin, etc.) were shown to be enhanced on the GO/PCL, compared to PCL control scaffolds. *In vivo* implantation of the conduits into a Sprague Dawley rat model (PCL, GO/PCL or autograph), showed that at 6 weeks post-surgery, the sciatic function index of the GO/PCL group was notably higher than the PCL group, but lower than the autograph group. Similar results were observed at 12 weeks, while at 18 weeks there was no significant difference between the GO/PCL and the autograph group.

Finally, 3D extrusion printing has also been used to fabricate intricately detailed scaffolds containing graphene (60 vol%) blended with polylactide-*co*-glycolide (PLG), forming printed features as small as 100 μm, with thicknesses of between <300 μm and >10 cm (123). The scaffolds were highly conductive (~800 S m^−1^), with biocompatibility testing with hMSCs showing that for *in-vitro* experiments in standard cell culture media and in the absence of neurogenic stimuli, the 3D printed scaffolds support the adhesion, viability, proliferation and neurogenic differentiation of hMSCs, with significant upregulation of glial and neuronal genes. *In-vivo* experiments showed the scaffolds to not elicit an inflammatory response, or suffer from fibrotic encapsulation, after 30 days post-surgery, highlighting the promise of this material for neural tissue engineering and regenerative medicine.

### Cardiac Tissue Engineering

The contractile behaviour of cardiac myocytes in cardiac tissue is reliant on the propagation of an electrical excitation signal through the electroconductive networks within the resident myocardium, producing the synchronous pulsatile pumping action required to drive blood through the heart and around the body. The reliance on this electrical signal transmittance through the native tissue presents an obvious opportunity for the use of electrically conductive biomaterials and networks to be used in cases where tissue damage (i.e., cardiac infarction) impedes the natural signal transmittance in the tissue, as well as assist in the repair and engineering of functional cardiac tissue. rGO – hydrogel composite materials have been shown to present good platforms for cardiac tissue engineering (82) and direct treatment of cardiac infarction (127). Shin et al. (82) developed an rGO-incorporated gelatin methacrylyol (GelMA) hybrid gel incorporating 0–5% rGO. The viability, proliferation and maturation of primary rat cardiomyocytes were enhanced on the rGO-GelMA scaffolds compared to the GelMA control, with cardiomyocytes on the rGO-GelMA scaffolds also showing stronger contractility and faster beating rates compared to those on the GelMA alone. An rGO coating on a porous collagen hydrogel was also shown to improve the viability of HUVEC cells above the collagen and TCP controls, however increasing the degree of modification above a 400 μg/ml rGO treatment solution resulted in a reduction in cell viability (127). Relative to the collagen control, the optimal electroactive rGO scaffolds (400 μg/ml) upregulated cardiac gene expression involved in electrical coupling (~2-fold increase in Cx43), muscle contraction and relaxation (~5-fold increase in troponin-T) and cytoskeleton alignment (~2-fold increase in actinin-4) after 7 days without electrical stimulation. The rGO-collagen scaffolds also demonstrated antibacterial properties against the pathogenic bacteria *Escherichia coli, Staphylococcus aureus and Streptococcus pyogenes*.

**Figure 8:**
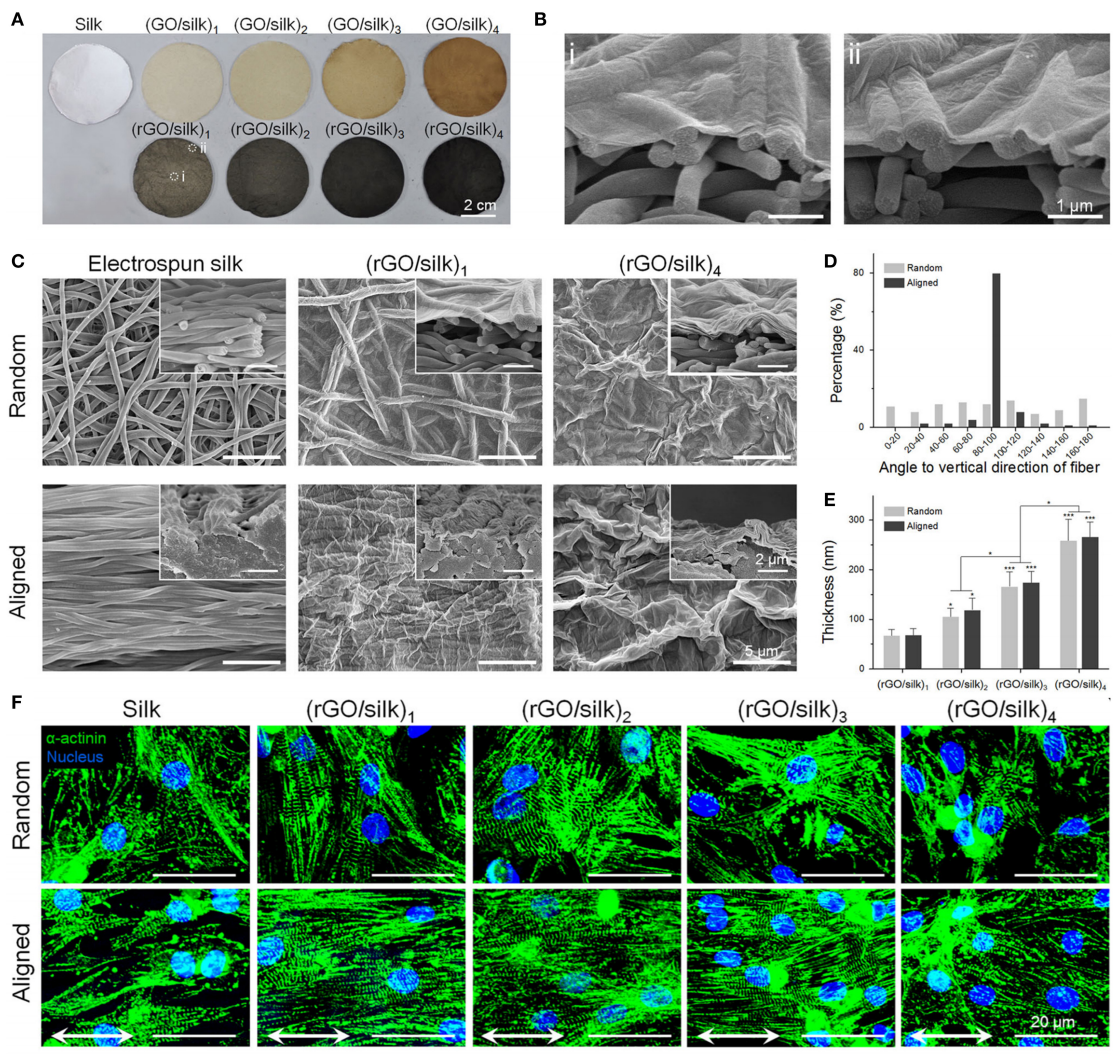
Photographic and morphologic characterizations on rGO/silk biomaterials. **(A)** Images of random silk, GO/silk, and rGO/silk materials. **(B)** Cross-sectional images of rGO/silk materials in the (i) central and (ii) marginal regions in **(A)** show no distinction in rGO coating thickness. **(C)** SEM images of the surface and cross-section of silk and rGO/silk scaffolds, showing an integrated rGO layer and a nanofibrous morphology that is controllable based on the coating thickness. **(D)** Fibre orientation distribution of random and aligned electrospun silk matrices. **(E)** rGO coating thickness shows a positive relationship with the GO doping mass. **(F)** Immunofluorescence show α-actinin expression for cardiomyocytes after day 7. Reproduced with permission from Zhao et al. (124). A significant difference is defined as *p < 0.05, **p < 0.01, ***p < 0.001.

Electrospinning has been employed to develop conductive scaffolds for cardiac tissue engineering, with rGO-polyurethane (126) and graphene-polycaprolactone (125) composites shown to produce nanofibrous scaffolds with good physicochemical and electrical properties, as well as excellent biocompatibility. Zhao et al. (124) used electrospinning to develop a nanofibrous silk-fibroin scaffold that was subsequently coated with GO using a vacuum filtration approach, with the adsorbed GO thereafter reduced rGO (Figure 8). This approach was used to develop electroactive rGO-silk fibroin scaffolds with random and aligned fibres, and when tested against primary rat cardiomyocytes, were shown to promote tissue formation and function. Cell alignment was promoted on the aligned electrospun scaffolds, with the formation of sarcomeric structures and gap junctions, and expression of α-actinin, cardiac troponin I (cTnI) and Cx43 upregulated on the rGO/silk scaffolds, relative to silk controls. The expression of cTnI and Cx43 were also enhanced by electrical stimulation (1 Hz, 5 V cm^−1^ for 4 days) of the cardiomyocytes on the rGO/silk scaffolds, thus presenting this material as an excellent candidate for further study for cardiac tissue regeneration applications.

An injectable GO-based electroactive hydrogel has also been investigated for application as an angiogenic gene delivery system for cardiac repair (128). This study proposed to use the injectable GelMA gel as a medium to deliver GO nanosheets functionalised with polyethylenimine (PEI) and vascular endothelial growth factor-165 (VEGF) pro-angiogenic gene for myocardial therapy. The injectable fGO_VEGF_/GelMA hydrogel was shown to be capable of transfecting myocardial tissues and inducing favourable therapeutic effects. A rat model with acute myocardial infarction was used, with the hydrogel shown to reduce scar tissue and enhance mitotic activities of endothelial cells, increasing myocardial capillary density at the injected region relative to controls.

### Bone Tissue Engineering

**Figure 9:**
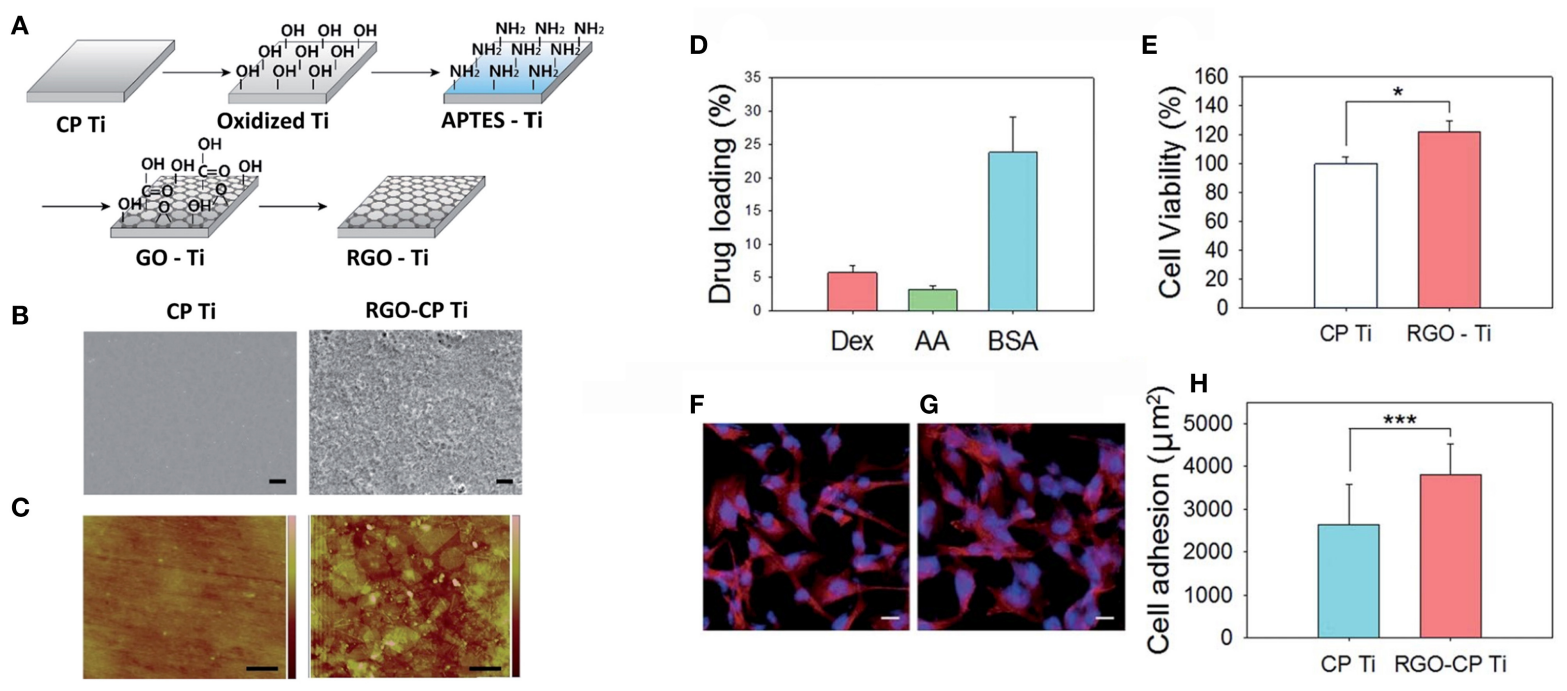
**(A)** Schematic illustration for the preparation of rGO-Ti. **(B)** SEM images of Ti and rGO-Ti (scale bar = 2 μm). **(C)** AFM images of Ti and rGO-Ti (scale bar = 500 nm). **(D)** Drug loading (%) on rGO-Ti. **(E)** Relative cell viability on Ti and rGO-Ti. Confocal microscopic images of F-actin stained preosteoblasts on **(F)** Ti and **(G)** rGO-Ti (scale bar = 25 μm). **(H)** Cell adhesion area determined from the region of F-actin fluorescence (*P ≤ 0.05 and ***P ≤ 0.001). Reproduced with permission from Jung et al. (130).

Graphene based biomaterials have shown great potential for bone tissue engineering, both through their ability to support and guide cellular interactions and behaviour, as well as through promoting mineralisation processes. Graphene and GO films on a PDMS substrate were tested for their ability to guide the differentiation of bone marrow mesenchymal stem cells towards an osteogenic lineage, relative to PDMS (129). The young's modulus of the graphene (~5 MPa) and GO (~7 MPa) were higher than PDMS (~3 MPa), with the contact angle of PDMS (117.70 ± 2.80) and graphene on PDMS (110.40 ± 1.20) significantly higher the GO on PDMS (38.30 ± 1.80). Cell proliferation was greater on graphene and GO compared to PDMS, which was also correlated with an increased adsorption of serum proteins on the graphene and GO (8 and 24% of total serum protein, respectively), with PDMS adsorbing <1%, after 1-day incubation. Graphene was shown to adsorb the highest amount of dexamethasone and β-glycerol-phosphate, compared to PDMS and GO, with cells cultured on graphene shown to present an enhanced osteogenic differentiation and deposited more minerals under chemical induction, compared to the other substrates. In another study, commercially pure titanium (CP Ti), a material commonly used in implants that interface directly with bone tissue, was modified with rGO and tested as an approach to accelerate bone regeneration and osseointegration (130). The viability and attachment of pre-osteoblast MC3T3-E1 cells was enhanced on the rGO modified Ti, compared to the Ti alone, demonstrating the ability for rGO to enhance cellular interactions. Furthermore, the rGO modified Ti was capable of adsorbing the osteogenic drugs dexamethasone and ascorbic acid, providing the ability to incorporate chemical cues into the material interface (Figure 9). *In-vivo* efficacy was determined through the implantation of dexamethasone modified rGO-Ti into the calvarial bone in the Sprague Dawley rat model, confirming effective bone regeneration and osteointegration of the implant.

3D porous scaffolds present an opportunity to regenerate and engineer structures of specific dimensions and size, providing the ability to reconstruct large defects within bone tissues. 3D porous composites composed of GO and nano-hydroxyapatite (nHA) (incorporating 20, 40 or 80% nHA) were fabricated using a self-assembly method, with 20% nHA -GO scaffolds found to significantly enhance the proliferation, alkaline phosphatase activity (ALP) and osteogenic gene expression (e.g., RUNX2, Col1A1, OCN) of rat bone mesenchymal stem cells (rBMSCs), relative to rGO alone and 40- and 80%-nHA scaffolds (133). *In-vivo* testing showed the 20% nHA-rGO scaffolds were capable of accelerating the healing for a 4 mm circular defect in the calvarial bone of a rabbit, relative to a rGO control scaffold, over a 6-week period. Porous GO-chitosan scaffolds containing 0, 0.5 or 3 wt% GO fabricated *via* freeze drying technique have also been shown to enhance the viability and proliferation of pre-osteoblast MC3T3-E1 cells, relative to chitosan alone (134), while GO sheets decorated with strontium nanoparticles (NPs) and blended with poly(ϵ-caprolactone) (PCL) have improved the biological activity of microporous scaffolds for bone tissue engineering (135). In the later study, the MC3T3-E1 cell proliferation and differentiation was greater on scaffolds containing the strontium NPs loaded GO sheets, compared to those without strontium NPs and PCL controls. The enhanced biological activity was attributed to the release of strontium ions from the strontium loaded GO sheets, with strontium known to activate osteoblast cells. Electrospun scaffolds formed from GO composites with starch (131) and PLA/nanohydroxyapatite (132) have additionally been shown to present good interfaces for osteoblast biocompatibility, as well as biomineralization processes.

### Skeletal Muscle Tissue Engineering

Ultrathin films of thermally reduced GO have been shown to improve the adhesion and spreading of C2C12 cells compared to GO films and a glass substrate (136). Electrical stimulation (8 V, 10 Hz frequency, and duration of 10 ms for 2 days) also improved differentiation on the thermally reduced GO vs. GO and glass, as determined using myogenic markers and proteins (increased myotube length and coverage area, and gene expression of αMRF4 and sarcomeric actin). The addition of uniaxially aligned topographical features through the crumpling of thin graphene films has also shown to provide cues for the alignment of C2C12 myoblasts and myotubes, with the differentiation and maturation of myotubes enhanced on the crumpled, relative to the flat, graphene films (137).

Electrically conductive rGO-polyacrylamide (PAAm) hydrogels, formed *via* a mild chemical reduction technique, were capable of presenting stiffness values similar to native muscle tissue (~50 kPa), and increased proliferation and differentiation of C2C12 myoblasts relative to GO/PAAm and PAAm hydrogels alone (139). Electrical stimulation (5 V, 10 ms duration, 1 Hz for 4 h every day) significantly increased myogenic gene expression for MyoD, myogenin and MHC, relative to unstimulated rGO/PAAm controls after 7 days culture. An aerogel composed of rGO and polydopamine (PDA) (rGO-PDA) has also been shown to present excellent biocompatibility, supporting C2C12 viability and spreading (140). A comparable electrical stimulation protocol as used above (5 V, 10 ms duration, 1 Hz for 4 h every day) revealed myogenin, MHC and α-actinin gene expression to be enhanced on stimulated vs. non-stimulated aerogels. Another study investigated the ability for GO films and GO-PCL electrospun fibrous meshes to guide the differentiation of hMSCs to the skeletal muscle cell lineage (138). Both the GO films and GO-PCL meshes supported the differentiation of MSCs towards skeletal myoblasts, as well as promoting cell alignment, with cell viability and proliferation highest on the GO-PCL after 11 days cell culture, relative to GO film, collagen and glass controls. Furthermore, myogenin expression increased more on thin GO sheets and GO-PCL than the collagen and glass controls, specific muscle antigens like MHC and dystrophin were expressed more intensely on the GO-PCL meshes compared to those on thin GO sheets or control substrates, indicating both the GO films and GO-PCL meshes present advantageous interfaces for the differentiation of MSCs towards a skeletal muscle lineage.

### Skin Tissue Engineering

rGO functionalised with the peptides glutamate and lysine were shown to be cytocompatible, degradable and bioactive, and to support the adhesion and growth of NIH-3T3 fibroblast cells and RAW macrophages, presenting favourable properties for skin tissue engineering applications (141). 3D graphene foams, as well as GO composites fabricated as nanofibrous mats and porous sponge-like scaffolds have also shown great potential for the engineering of skin tissue layers. Lee et al. (142) fabricated electrospun mats composed either of GO-PLGA or GO-PLGA/collagen (Col). Incorporation of GO and GO/Col into the PLGA scaffold increased material hydrophilicity (143.5 ± 4.8°, 127.9 ± 2.7°, and 54.6 ± 3.7° for PLGA, GO-PLGA, and GO-PLGA/Col, respectively), with initial cell attachment of human dermal fibroblasts significantly greater on the GO-PLGA and GO-PLGA/Col samples, relative to the PLGA control. Graphene foams (GFs), fabricated through CVD deposition of graphene on porous Ni templates, were demonstrated to support the adhesion and growth of MSCs, with cell viability not affected and proliferation significantly increased after 5 days culture on the GFs, relative to TCPs control (143). GFs loaded with MSCs were implanted into a rat model, and their ability to influence wound healing and regenerate dermal tissue assessed. Microscopic observation revealed the GFs loaded with MSCs to obviously enhance wound closure, relative to the GFs alone or no scaffolds (control). Furthermore, the dermis regenerated in the presence of the GFs with MSCs was thicker and more complex in structure after 14 days, with the presence of the MSCs in the transplanted scaffolds inducing a significant upregulation in vascular endothelial growth factor (VEGF) and basic fibroblast growth factor (bFGF). Nyambat et al. (144) formed a genipin crosslinked ECM sponge scaffold, using ECM derived from decellularized adipose stem cell (ASC) sheets, and incorporating different amounts of GO in order to improve ECM sponge mechanical properties and degradability. Scaffolds incorporating 20 μg/ml GO presented excellent biocompatibility (cell adhesion, proliferation, and migration) when tested using L929 fibroblast cells, with *in-vivo* experiments revealing the sponges to demonstrate appropriate biodegradation with limited inflammatory response when implanted in the subcutaneous tissue of a rat model over a 4-week period.

### Cartilage Tissue Engineering

The ability for graphene and GO to effectively interface with bioactive compounds and proteins has been exploited as an approach to enhance the biofunctionality and biointerfacing of graphene and graphene-biocomposites for cartilage regeneration. Graphene, or GO flakes were incubated overnight in growth factors, and subsequently mixed with MSCs to form a cell – graphene biocomposite (145). Previous work by the authors had demonstrated the ability for graphene and GO to concentrate growth factors *via* their surface adsorption through π-π stacking, electrostatic and hydrogen bonding (129). Here, the incorporation of graphene and GO were positively correlated with an increase in chondrogenic cell differentiation, although increasing graphene and GO above a certain level was seen to result in cytotoxic properties. In another study (148) GO nanoparticles were loaded with the bone morphogenetic protein (BMP-7), a protein important in cartilage homeostasis and repair, and thereafter blended with collagen and chitosan solution to form a 3D printable hydrogel. BMP-7 release from the hydrogels incorporating the modified GO revealed release over a 30-day period, providing extended chondrocyte protection, with *in-vivo* experiments using printed scaffolds confirming the potential of this approach.

The fabrication of porous composite scaffolds has also shown promise for cartilage tissue engineering, with incorporation of up to 0.3% GO in a porous chitosan scaffold shown to increase the proliferation of human articular chondrocytes over a 14 day period (146). In another study, a porous hybrid scaffold containing methacrylated chondroitin sulphate (CSMA), poly(ethylene glycol) methyl ether-ϵ-caprolactone-acryloyl chloride (PECA) and GO (3 wt%) was evaluated for its capacity to promote the regeneration of cartilage tissue (147). In an attempt to best mimic the properties of the extracellular matrix, a scaffold with approximate pore size, porosity, swelling ability, compression modulus and conductivity were tailored by varying the CSMA:PECA ratio. *In vitro* studies using 3T3 cells demonstrated scaffold biocompatibility, and *in vivo* studies involving implantation into a full thickness articular cartilage defect in a rabbit model showed improved chondrocyte morphology, integration, continuous subchondral bone, and much thicker newly formed cartilage compared with control groups. A 3D printable photopolymerisable hydrogel ink composed of GelMA, poly (ethylene glycol) diacrylate (PEGDA) has also been developed to promote the chondrogenic differentiation of MSCs (52). The incorporation of GO in the scaffold promoted the chondrogenic differentiation of MSCs relative to controls without GO, as well as increased cell adhesion and proliferation. This was proposed to be driven through the loading of various bioactive components (i.e., growth factors, proteins) in the media to the GO surface, resulted by strong π-π bonding and electrostatic interactions. Critically, the GelMA-PEGDA-GO scaffolds greatly enhanced glycosaminoglycan, total protein and collagen levels after GO induced chondrogenic differentiation of MSCs, highlighting the potential of this printable material for cartilage tissue engineering.

## Emerging Opportunities for Organic Conductors in Tissue Engineering

There is ample evidence to suggest that the use of organic conductors to control the proliferation and differentiation of living cells has provided significant advances. One must consider then how such technologies may be translated into practical application. The obvious divide is *in vitro* vs. *in vivo*. *In vitro* platforms could serve as tools to prime cellular containing constructs prior to implantation. This requires further study to investigate the effect of such pioneered constructs on performance in animal models. It seems the tools are available to pursue such investigations. *In vivo* – the use of OCPs to stimulate constructs after implantation presents more technological challenges. The OCP electrodes should ideally be biodegradable, to allow them to exit the treatment site once the mission is completed. While some studies (173, 174) into biodegradable electrodes exist they are limited. The development of wireless, implantable stimulation systems is also critical to *in vivo* deployment. There have been marked advances in this area in recent year including very small RF transmission systems (175, 176) and indirect methods that use ultrasound to deliver (piezo) electrical effects (177–180). Neither have been coupled to OCPs to date. *In vivo* – one should also consider regulatory issues and use of simpler materials (graphenes) may outplay the use of the more complicated OCPs in this regard.

## Author Contributions

BM, PM, JF, and GW conceived the review topic and designed the manuscript structure. BM and PM wrote the first draught of the review. JF and GW provided corrections and additions. All authors contributed to the article and approved the submitted version.

## Conflict of Interest

The authors declare that the research was conducted in the absence of any commercial or financial relationships that could be construed as a potential conflict of interest.
